# Genetic identification of acetyl-CoA synthetases involved in acetate activation in *Haloferax mediterranei*

**DOI:** 10.1128/aem.01843-24

**Published:** 2024-12-31

**Authors:** Ruchira Mitra, Yang Xu, Lin Lin, Jing Guo, Tong Xu, Mengkai Zhou, Feng Guo, Hao Li, Hua Xiang, Jing Han

**Affiliations:** 1 State Key Laboratory of Microbial Resources, Institute of Microbiology, Chinese Academy of Sciences 85387 , Beijing, Beijing, China; 2 International College, University of the Chinese Academy of Sciences 617858 , Beijing, Beijing, China; 3 College of Life Science, University of the Chinese Academy of Sciences 617066 , Beijing, Beijing, China; 4 College of Life Science, Yunnan University 12635 , Kunming, Yunnan, China; 5 College of Life Science, Shandong Normal University 47856 , Jinan, Shandong, China; Kyoto University, Kyoto, Japan

**Keywords:** haloarchaea, AMP-acetyl-CoA synthetase, ADP-acetyl-CoA synthetase, acetate activation

## Abstract

Acetate/acetyl-CoA interconversion is an interesting metabolic node, primarily catalyzed by a set of various enzymes in prokaryotes. *Haloferax mediterranei* is a promising haloarchaeaon, capable of utilizing acetate as a sole carbon source for biosynthesis of high value-added products. Here, we have reported the key enzymes that catalyzed acetate activation in *H. mediterranei*. Based on bioinformatic and transcript analysis, thirteen possible candidate genes were screened. Simultaneous deletion of eleven genes led to a mutant strain (named as Δ11) that failed to grow on acetate. Gene complementation in Δ11 revealed six AMP-ACS (encoded by HFX_0870, HFX_1242, HFX_1451, HFX_6342, HFX_5131, and HFX_1643) and one ADP-ACS (encoded by HFX_0998) to be functional in acetate activation. Furthermore, heterologous expression of ADP-ACS genes from *Haloarcula hispanica* and *Haloferax volcanii* catalyzed acetate activation in Δ11. Subsequently, it was observed that, deletion of the six AMP-ACS genes in *H. mediterranei* ceased the cell growth of the resulting mutant (Δ6AMP-ACS) on acetate. An *in vivo* function of ADP-ACS in acetate activation could be excluded since ADP-ACS was downregulated on acetate. However, plasmid-based overexpression of ADP-ACS enabled Δ6AMP-ACS to grow on acetate, even better than the parent strain. Thus, it can be inferred that native ADP-ACS with low expression level was unable to mediate cell growth of Δ6AMP-ACS on acetate. This is the first genetic evidence exhibiting that overexpression of haloarchaeal ADP-ACS catalyzed acetate activation *in vivo*. Collectively, this is a comprehensive study of acetate activation in *H. mediterranei,* and the current findings would surely enrich the understanding of acetate metabolism in archaea.

**IMPORTANCE:**

Owing to the high demand and supply challenge of glucose, acetate might be considered a potential alternative carbon source for microbial growth and fermentation. *Haloferax mediterranei* is capable of utilizing acetate as a carbon source for growth and subsequent value-added product synthesis. Thus, it is essential to identify the genes responsible for acetate utilization in *H. mediterranei*. As per available literature, haloarchaeal ADP-forming acetyl-CoA synthetase (APD-ACS) catalyzes the reversible conversion of acetate to acetyl-CoA *in vitro*. However, *in vivo*, acetate activation and acetate formation are catalyzed by AMP-forming acetyl-CoA synthetase (AMP-ACS) and ADP-ACS, respectively. In this study, we have identified six AMP-ACS enzymes that catalyzed acetate activation in *H. mediterrane*i. Deletion of these six genes abolished the growth of the resulting mutant (Δ6AMP-ACS) in acetate medium. The natively expressed ADP-ACS was unable to mediate its acetate activation *in vivo*. Interestingly, an artificial system based on plasmid overexpression of ADP-ACS in Δ6AMP-ACS restored its growth on acetate. This finding suggested that native ADP-ACS was unable to catalyze acetate activation in *H. mediterranei* due to its low expression level. Together, our study explored the acetate activation in *H. mediterranei,* and the obtained results would enrich the knowledge of acetate metabolism in archaea. Furthermore, the information offered in this study would benefit the improvement of acetate utilization in haloarchaea for value-added product synthesis.

## INTRODUCTION

Acetate, a two-carbon molecule, is a readily available, cost-effective, and promising multifaceted substrate for microbial fermentation (1). When acetate is used as a carbon source for microbial fermentation, it is first transported into the cell by the acetate transporter, followed by its activation to acetyl-CoA (2). Usually, in all the three domains (bacteria, archaea, and eukarya), acetate activation to acetyl-CoA is carried out by the AMP-forming acetyl-CoA synthetase (AMP-ACS) pathway (3). In this process, acetate is first activated to acetyl-adenosine monophosphate (acetyl-AMP) using ATP. Then, acetyl-AMP forms a thioester bond with coenzyme A to generate acetyl-CoA, while releasing pyrophosphate (PP_i_) and AMP (acetate +ATP + CoA → Acetyl-CoA +AMP + P_Pi_) (4). However, in certain bacteria, variations in the acetate activation enzymes do exist. For instance, although the acetate kinase/phosphotransacetylase (AK/PTA) pathway primarily operates in the direction of acetyl-CoA to acetate conversion in bacteria, acetate activation in *Corynebacterium glutamicum* is mediated by the AK/PTA pathway (5). Here, acetate is first converted to acetylphosphate, catalyzed by AK, which is further converted to acetyl-CoA by PTA. Furthermore, acetate activation in *Escherichia coli* involves both the AMP-ACS and AK/PTA pathways, depending upon the acetate concentration (6). The AMP-ACS pathway operates at low concentrations, whereas AK/PTA operates at a high acetate concentration in *E. coli*. Additionally, in few bacteria including *Acetobacter aceti* and sulfate reducers, the acetate to acetyl-CoA conversion is catalyzed by succinyl-CoA:acetate CoA-transferase (7, 8).

Acetate activation in archaea is mostly catalyzed by AMP-ACS (3, 9), except for methanogenic archaea *Methanosarcina* spp., which involves the AK/PTA pathway (10). Several haloarchaea (including *Natronomonas pharaonis*, *Natrialba magadii*, *Natrialba wudunaoensis*, *Natrialba chahannaoensis*, *Haloferax volcanii*, *Haloarcula marismortui*, *Halorubrum saccharovorum*, and *Halococcus saccharolyticus*) are known to be capable of utilizing acetate as a carbon source (9, 11–14). In *H. volcanii*, acetate activation catalyzed by AMP-ACS has been characterized both genetically and kinetically (9, 15). Haloarchaeal AMP-ACS enzymes are acetate-inducible. They exhibit high substrate affinity for acetate, which allows them to activate a low concentration of acetate present in its surrounding (9). Unlike bacteria, the acetate formation in haloarchaea is catalyzed by ADP-ACS instead of the AK/PTA pathway (15, 16). ADP-ACS is often known as the haloarchaeal one-enzyme counterpart of the bacterial AK/PTA couple (13). It was first characterized in hyperthermophilic euryarchaeon *Pyrococcus furiosus*; later it has been characterized in haloarchaea *H. marismortui* and *H. volcanii* (15–17). Interestingly, haloarchaeal ADP-ACS catalyzes the reversible conversion of acetyl-CoA to acetate (acetyl-CoA +ADP + P_i_ ⇔ acetate +ATP + CoA) *in vitro* (15). However, *in vivo*, ADP-ACS is not considered to be involved in acetate activation; rather it participates in acetate overflow, as evidenced in *H. volcanii*. Deletion of four functional AMP-ACS genes abolished cell growth of *H. volcanii* on acetate, indicating non-involvement of ADP-ACS in acetate activation *in vivo* (15). Furthermore, deletion of the ADP-ACS gene reduced the acetate overflow in *H. volcanii* when cultured in high-glucose medium (15). Thus, based on the available literature, it is evident that acetate activation in haloarchaea is catalyzed by AMP-ACS and acetate formation is catalyzed by ADP-ACS.

*Haloferax mediterranei* is capable of utilizing acetate for biosynthesis of polyhydroxyalkanoates (PHA) and other value-added products such as carotenoids (18, 19). The already available complete genome sequence and the efficient gene manipulation system have made *H. mediterranei* a promising model organism for studying haloarchaeal physiology and metabolism (20, 21). However, its acetate activation remains unclear. In this context, the present study comprehensively explored the acetate activation in *Haloferax mediterranei* by the genetic approach. In total, thirteen possible candidate genes for acetate activation were screened by bioinformatics and transcript analyses. Growth of *H. mediterranei* was completely abolished when eleven genes were simultaneously knocked out. The functional acetate activation genes were then identified by gene complementation and were further validated by their gradual deletion in *H. mediterranei*. Finally, we have identified six functional AMP-ACS involved in acetate activation in *H. mediterranei*. Notably, this is the first genetic evidence showing that ADP-ACS can catalyze acetate activation *in vivo* in haloarchaea during its plasmid-based overexpression.

## MATERIALS AND METHODS

### Strains, medium, and culture conditions

Strains used in this study are enlisted in Table S1. *Escherichia coli* JM109 and *E. coli* JM 110 were used for plasmid construction and elimination of methylated plasmids, respectively. Both *E. coli* strains were cultivated in Luria–Bertani medium at 37°C (22). When required, ampicillin was added at a concentration of 100 µg/mL. *H. mediterranei* DF50ΔEPS, an uracil-auxotrophic strain (*pyrF* gene knockout) with a deleted EPS gene cluster, was used as the parent strain for gene knockout (21, 23). For counter-selection of knockout mutants, 50 µg/mL uracil and 250 µg/mL 5-fluoroorotic acid (5-FOA) were used. For seed culture preparation, DF50ΔEPS and its knockout mutants were grown in AS-168 liquid medium with 50 µg/mL uracil (AS-168U) at 37°C (24). The seed culture was then inoculated in the fermentation medium comprising NH_4_Cl (2 g/L), MgSO_4_∙7H_2_O (20 g/L), KCl (2 g/L), NaCl (110 g/L), PIPES (15 g/L), yeast extract (0.5 g/L), CaCl_2_ (0.5 g/L), KH_2_PO_4_ (37.5 mg/L), uracil (50 g/ml), and glucose (7.2 g/L = 0.04 M) or acetate (9.84 g/L = 0.12 M or 3.28 g/L = 0.04 M, as required). For cultivation of the complementation or overexpression strains, a similar medium excluding yeast extract and uracil was used. *H. volcanii* H1424 was used for protein expression and was grown in the Hv-YPC medium containing the yeast extract (5 g/L), peptone (1 g/L), and casamino acids (1 g/L) at 45°C, supplemented with 50 µg/mL uracil and 20 µg/mL thymidine (25).

### Construction of plasmids and mutants

All plasmids and primers used in this study are summarized in Tables S1 and S2, respectively. Plasmids for gene deletion were constructed based on the suicide plasmid pHFX (21) or pHX-B60 (22). All plasmids for gene complementation and gene overexpression were constructed based on the shuttle plasmid pWL502 (24). The transformations of *H. mediterranei* and *H. volcanii* were performed by the polyethylene glycol (PEG)-mediated transformation method (26). Gene mutant construction and verification were performed by pop-in/pop-out method and PCR, respectively, as reported previously (21).

### Cell growth determination

The growth of *H. mediterranei* strains was quantitatively analyzed by measuring the absorbance of the liquid culture at 600 nm using a Beckman Coulter DU800 spectrophotometer (Jersey City, NJ, USA). Briefly, the *H. mediterranei* strains were grown in AS-168U liquid medium for 24 hours. The seed culture was then inoculated into 10 mL of the fermentation medium. OD_600_ was measured at an interval of 24 hours for 5 days. The initial OD_600_ was adjusted to 0.1. All the growth comparison experiments were performed in three replicates.

### Acetate quantification by HPLC

The acetate concentration in the supernatant of the fermentation culture was quantified using a 1260 infinity high-performance liquid chromatography (HPLC) system (Agilent, USA) equipped with a variable wavelength detector (Agilent, USA). The Aminex HPX-87H column (7.8 × 300 mm, Bio-Rad, USA) was used for separation of the organic components in the supernatant. The samples were filtered using a 0.22-µm filter and eluted with 5.0 mM sulfuric acid at a flow rate of 0.5 mL/min. The total analysis time for a single injection was 20 minutes. Finally, the eluents were monitored at a wavelength of 210 nm.

### RNA extraction, qRT-PCR, and transcriptome analysis

For qRT-PCR, *H. mediterranei* DF50ΔEPS cells were grown in the fermentation medium containing 0.12 M acetate or 0.04 M glucose as the sole carbon source up to the exponential phase. The cells were harvested for total RNA extraction using TRIzol reagent (Invitrogen, USA) according to the manufacturer’s instruction. About 10 µg of total RNA was digested using the TURBO DNA-free Kit (Thermo Fischer Scientific, USA) to remove DNA from samples. Then, cDNA was synthesized from the DNA-free RNA samples using random hexamer primers and Moloney Murine Leukemia Virus Reverse Transcriptase (MMLV-RT) (Promega, USA). All real-time PCRs were performed using the KAPA^TM^ SYBR® Fast qPCR Kit (KAPA Biosystems, USA) and analyzed by ViiA 7 Real-Time PCR System (Applied Biosystems, Inc., USA). The relative fold change of target gene expression was calculated by the 2_ΔΔCT (where CT is threshold cycle) mathematical model using 7S RNA as an internal standard (27). The primers used in this study are listed in Table S2. qRT-PCR was performed in three replicates.

For transcriptome analysis, the *H. mediterranei* mutant strain was cultured in the fermentation medium containing 0.12 M acetate or 0.04 M glucose up to its exponential phase, followed by total RNA extraction from harvested cells. After checking the RNA purity and integrity, 3 µg of RNA was used to construct a library for strand-specific transcriptome sequencing using the NEB Next UltraTM RNA Library Prep Kit for Illumina (NEB, USA). After cluster generation and sequencing using a HiSeq T2500 (Illumina, USA) by Novogene Co., Ltd. (Beijing, China), clean data were obtained by removing reads containing the adapter or poly(N) and low-quality reads. Then, the read numbers mapped to each gene were counted by HTSeq, version 0.6.1. The expected number of fragments per kilobase of transcript per million mapped reads (FPKM) of each gene was further calculated and used to present the change in the expression of each gene. Differential expression analysis was performed using the DEGSeq R package (1.20.0) (23). Three biological replicates were set for each group. The transcriptome data of *H. mediterranei* Δ7 were deposited in the China National Microbiology Data Center under the accession number NMDC60146101.

### Proteomic analysis

For proteomic analysis, *H. mediterranei* DF50ΔEPS cells were cultivated in the fermentation medium containing 0.12 M acetate or 0.04 M glucose as the sole carbon source up to their exponential phase. Cells were harvested and washed with PBS buffer. The protein was extracted by treating the cells with SDT lysis buffer (4% SDS, 100 mM Tris–HCl, 100 mM dithiothreitol, pH 7.6), followed by ultrasonication. Cellular debris were removed after centrifugation, and the supernatant was quantified using the BCA Protein Assay Kit (Beyotime, China). Then, the protein was subjected to trypsin digestion by using the FASP method (28). LC-MS/MS analysis was performed on an Orbitrap Astral mass spectrometer coupled with Vanquish Neo UHPLC system (Thermo Fisher Scientific). Peptides were loaded into a column (50 cm Low-Load µPAC Neo HPLC Column，Thermo Scientific) at a flow rate of 2.2 µL/min. The eluted peptides were subjected to DIA MS (data-independent acquisition mass spectrometry) analysis on the Orbitrap Astral mass spectrometer. The DIA MS data were analyzed using DIA-NN 1.8.1 software (29). Bioinformatic analysis was carried out with Microsoft Excel and *R* statistical computing software. Differential expression was represented as log_2_(fold change) with a *p*-value less than 0.05 considered significant. Three biological replicates were set for each group. The proteome data of *H. mediterranei* DF50ΔEPS were deposited in the China National Microbiology Data Center under the accession number NMDCM0000257.

### Statistical analysis

The results were presented as the mean ± standard deviation (SD) of three independent replicates. Significant differences among groups were identified by one-way analysis of variance (ANOVA), with statistical significance defined at a *p-*value of < 0.05.

## RESULTS

### Screening of potential candidate genes encoding acetyl-CoA synthetase

The whole-genome sequence of *H. mediterranei* revealed the presence of six candidate genes encoding AMP-ACS and one candidate gene encoding ADP-ACS (Table 1). The six putative AMP-ACS proteins were 535 to 664 amino acids long, and their molecular mass was calculated in the range from 59.68 kDa to 74.36 kDa. Among the six encoding genes, HFX_0870, HFX_1242, HFX_1643, and HFX_2150 were located on the chromosome and HFX_5129 and HFX_5131 were located on the pHM300 megaplasmid. All the six putative AMP-ACS proteins exhibited homology with each other (mostly ranged from 28.54 to 86.86% identity) (Table 2). Interestingly, genetic organization of HFX_5129 and 5131 presented high similarity with that of *H. volcanii* (Fig. S1A). HFX_5133, annotated as sodium/solute symporter, showed 95.6% homology with the reported acetate transporter (ActP in *H. volcanii*). Thus, HFX_5133 might be speculated as a candidate acetate transporter gene in *H. mediterranei*. The putative ADP-ACS protein encoded by HFX_0998 was a 697-amino acid-containing polypeptide with a calculated molecular mass of 74.47 kDa. It showed no significant similarity with the above six putative AMP-ACS proteins. Furthermore, a putative PTA protein encoding gene, HFX_0997, was located adjacent to HFX_0998 on the chromosome. Such a genetic organization of ADP-ACS and PTA-encoding candidate genes was widespread among haloarchaea, including *H. volcanii* and *Haloarcula hispanica* (Fig. S1B).

**TABLE 1:** Possible candidate genes involved in acetate metabolism of *H. mediterranei*

Name	Gene number	Location	Encoded protein	Amino acid number	Mw (kDa)
AMP-ACS	HFX_0870	Chromosome	AMP-forming acetyl-CoA synthetase	662	74.36
	HFX_1242			535	59.68
	HFX_1643			664	74.03
	HFX_2150			554	60.85
	HFX_5129	pHM300		651	71.63
	HFX_5131			664	73.98
ADP-ACS	HFX_0998	Chromosome	ADP-forming acetyl-CoA synthetase	697	74.48
PTA	HFX_0997	Chromosome	Putative phosphate acetyltransferase	364	38.8
Acetate transporter	HFX_5133	pHM300	Sodium/solute symporter	549	58.13

**TABLE 2:** Thirteen possible candidate genes for acetate activation and their amino acid sequence homology^*a*^

HFX number	_0870	_1242	_1451	_1643	_1837	_1860	_2150	_4020	_5129	_5131	_5190	_6342	_0998
_0870	100	30.62	26.05	38.54	23.12	–	32.37	23.99	64.94	86.86	–	23.33	–
_1242		100	30.53	32.96	33.79	28.33	41.18	31.73	28.54	28.99	–	32.68	–
_1451			100	24.08	34.18	–	30.14	32.24	26.57	26.80	–	35.37	–
_1643				100	21.92	–	32.99	28.19	38.10	37.87	–	24.81	–
_1837					100	–	32.58	33.02	24.63	23.94	–	59.85	–
_1860						100	–	–	–	–	–	–	–
_2150							100	33.53	32.74	33.10	–	32.09	–
_4020								100	25.77	25.13	–	35.17	–
_5129									100	65.40	–	26.01	–
_5131										100	–	23.15	–
_5190											100	–	–
_6342												100	–
**_**0998													100

^*a*^ The amino acid sequence homology is represented in percentage (%).

^*b*^ - represents no homology

### Transcriptional analysis of the six AMP-ACS genes and one ADP-ACS gene

**Fig 1:**
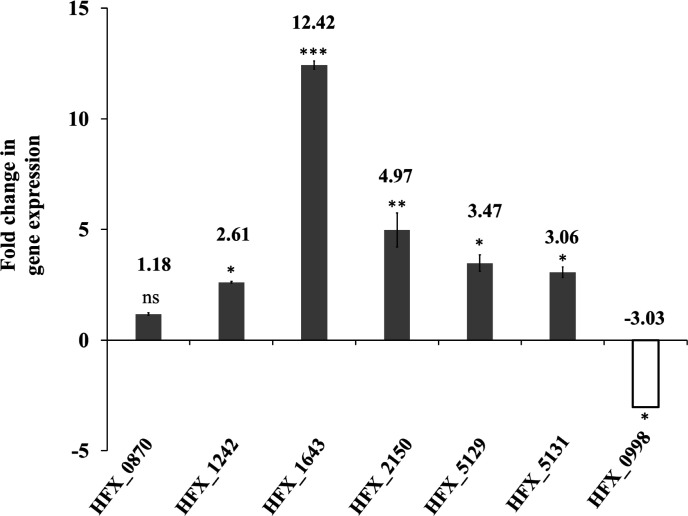
Upregulation of AMP-ACS genes and downregulation of ADP-ACS genes in *H. mediterranei* on acetate compared to glucose. qRT-PCR analysis for fold change in mRNA expression levels of the six putative AMP-ACS encoding genes, namely, HFX_0870, HFX_1242, HFX_1643, HFX_2150, HFX_5129, and HFX_5131 (represented by the gray column) and one putative ADP-ACS-encoding gene, namely, HFX_0998 (represented by white column) of *H. mediterranei*. DF50ΔEPS cultured to the exponential growth phase on 0.12 M acetate or 0.04 M glucose was used for qRT-PCR analysis. ns represents non-significant (*p-* value > 0.05), ** represents *p*-value < 0.01, and *** represents *p*-value < 0.005. Data are expressed as mean ± SD, *n* = 3.

To unravel which putative AMP-ACS enzyme catalyzed acetate activation in *H. mediterranei*, the transcript level of the respective candidate genes in acetate-grown cells was analyzed and compared with that of glucose-grown cells. qRT-PCR was performed, with the total RNA extracted from cells harvested during the exponential growth phase. As shown in Fig. 1, the mRNA expression level of the six putative AMP-ACS proteins was upregulated in acetate compared to glucose medium. The respective transcript levels of HFX_1643, HFX_2150, HFX_5129, HFX_5131, HFX_1242, and HFX_0870 were 12.42, 4.97, 3.47, 3.06, 2.61, and 1.18-fold higher in acetate compared to glucose, respectively. This observation indicated that the six AMP-ACS encoding genes were induced by acetate, and thus they might be involved in acetate metabolism. Furthermore, comparison of the transcript level of the putative ADP-ACS encoding gene in acetate- and glucose- grown cells revealed that its mRNA expression was ~threefold downregulated in acetate, therefore indicating glucose-specific upregulation of putative ADP-ACS. This result was reasonable as ADP-ACS is reported to catalyze acetate formation from acetyl-CoA during glucose fermentation in haloarchaea.

### Genetic characterization of AMP-ACS and ADP-ACS

**Fig 2:**
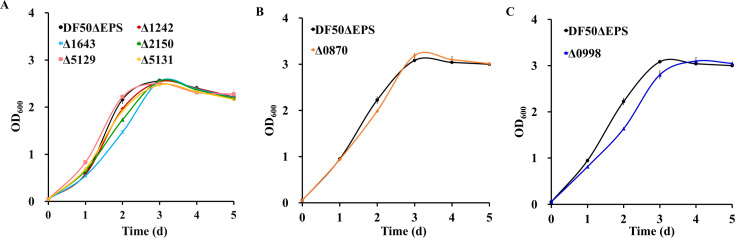
Effect of single *acs* gene knockout on cell growth of *H. mediterranei* on 0.12 M acetate. (**A**) Growth analysis of Δ1242, Δ1643, Δ2150, Δ5129, and Δ5131. (**B**) Growth analysis of Δ0870. For A and B, the fermentation time lasted for 5 days, and they were cultured in two different batches. Δ1242 (red line), Δ1643 (blue line), Δ2150 (green line), Δ5129 (pink line), Δ5131 (yellow line), and Δ0870 (orange line) represent the single mutant of six putative AMP-ACS genes, namely, HFX_1242, HFX_1643, HFX_2150, HFX_5129, HFX_5131, and HFX_0870. (**C**) Growth analysis of Δ0998 (mutant of the putative ADP-ACS encoding gene, HFX_0998) (dark blue line). DF50ΔEPS (black line) represents the positive control. Data are expressed as mean ± SD, *n* = 3.

Based on the transcriptional analysis, it was speculated that one or more among the six putative AMP-ACS proteins was involved in the acetate activation process. To study their physiological function, the corresponding genes were individually knocked out in *H. mediterranei* DF50ΔEPS. Six knockout plasmids were constructed and transformed into DF50ΔEPS, resulting in six single knockout mutants (Table S1). The mutants were cultivated in liquid medium containing 0.12 M acetate as the sole carbon source. All the six AMP-ACS mutants and the control DF50ΔEPS reached a similar OD_600_ value at the stationary phase (Fig. 2A and B). Δ1643, Δ2150, Δ5131, and Δ1242 exhibited a slightly reduced growth only during the exponential phase, whereas Δ5129 and Δ0870 showed no changes in their growth pattern (Fig. 2A and B). Possibly, the acetate activation activity of the deleted AMP-ACS gene was functionally replaced by other AMP-ACS paralogs, indicating that two or multiple AMP-ACS enzymes were involved in acetate activation in *H. mediterranei*.

According to the available literature, haloarchaeal ADP-ACS catalyzed the reversible conversion of acetyl-CoA to acetate *in vitro* (Fig. S2A); however, it was functionally involved only in acetate formation *in vivo*. In this study, HFX_0998 of *H. mediterranei* was heterologously expressed in *H. volcanii* H1424, and the purified protein exhibited a matching score of 11,177 and 72% sequence coverage with the ADP-ACS amino acid sequence. When subjected to biochemical assays, ADP-ACS-His_6_ catalyzed acetate formation from acetyl-CoA and *vice versa* (Fig. S2), which was consistent with the previous findings (15, 16). However, glucose-specific upregulation of HFX_0998 in transcriptomic analysis indicated its functional involvement in acetate formation *in vivo*. To further verify the function of ADP-ACS in *H. mediterranei*, HFX_0998 was knocked out in DF50ΔEPS, generating the Δ0998 mutant. The role of ADP-ACS in acetate overflow from 55 mM glucose was then analyzed (Fig. S3). Both DF50ΔEPS and Δ0998 exhibited a similar growth pattern on glucose. Glucose utilization was somewhat rapid in DF50ΔEPS compared to Δ0998. Strikingly, acetate accumulation was not detected for both DF50ΔEPS and Δ0998. Thus, involvement of ADP-ACS in acetate formation remained uncertain in *H. mediterranei,* and further clarification is essential. Following this, the function of ADP-ACS in acetate activation *in vivo* was also analyzed. Growth of Δ0998 on 0.12 M acetate was compared with that of DF50ΔEPS, and a slight reduction during the exponential phase was observed, indicating possible participation of HFX_0998 in acetate activation (Fig. 2C). Taken together, the possible involvement of the haloarchaeal ADP-ACS enzyme in acetate activation *in vivo* was unexpected and thus necessitated further experiments to substantiate this finding.

### Acetate activation in *H. mediterranei* is catalyzed by multiple ACS enzymes

**Fig 3:**
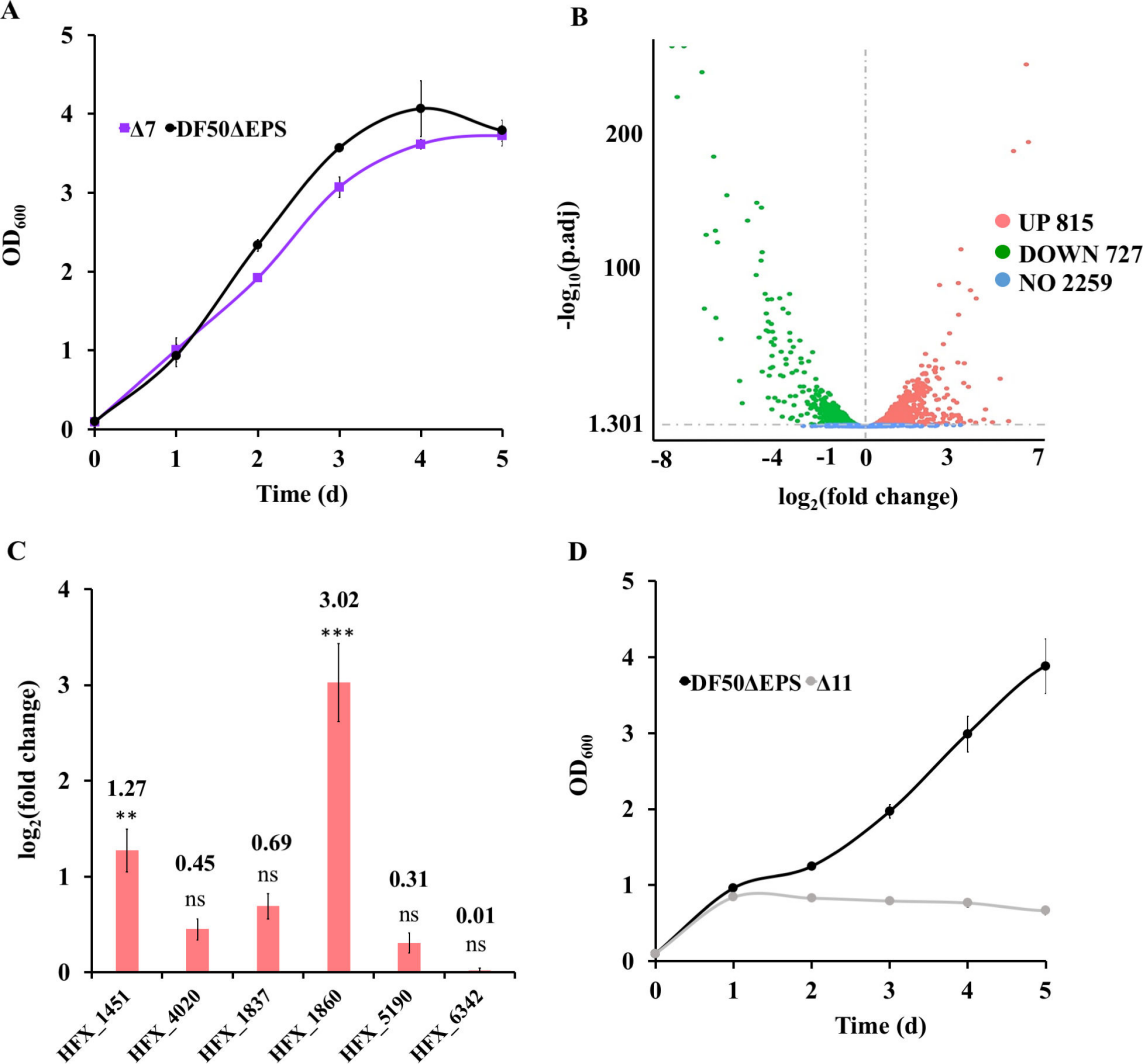
*H. mediterranei* Δ11 lost its ability to grow on acetate. (**A**) Growth analysis of Δ7 on 0.12 M acetate. Δ7 (violet line) is the multiple *acs* mutant of DF50ΔEPS with the six putative AMP-ACS genes (HFX_0870, HFX_1242, HFX_1643, HFX_2150, HFX_5129, and HFX_5131) and one ADP-ACS gene (HFX_0998) knocked out. (**B**) Volcano plot of differentially expressed genes of Δ7 in 0.12 M acetate compared to 0.04 M glucose. Pink circles represent upregulated genes, green circles represent downregulated genes, and blue circles represent genes with no change in expression. (**C**) Changes in the expression level of potential AMP-ACS candidate genes in Δ7, represented as log_2_(fold change) of gene expression in 0.12 M acetate compared to 0.04 M glucose. The candidate genes are annotated as following. HFX_1451, medium-chain-fatty-acid-CoA synthetase; HFX_4020, long-chain acyl-CoA synthetase; HFX_1837, medium-chain acyl-CoA synthetase; HFX_1860, CoA transferase; HFX_5190, putative acyl-CoA transferase; HFX_6342, medium-chain acyl-CoA synthetase. ns represents non-significant (*p*-value > 0.05), ** represents *p*-value < 0.01, and *** represents *p*-value < 0.005. (**D**) Growth analysis of Δ11 (gray line) on 0.12 M acetate. Δ11 represents the mutant of eleven genes, which includes ten possible AMP-ACS genes (HFX_0870, HFX_1242, HFX_1451, HFX_1643, HFX_1837, HFX_2150, HFX_4020, HFX_5129, HFX_5131, and HFX_6342) and one ADP-ACS gene (HFX_0998). DF50ΔEPS (black line) represents the positive control (A and D). Data are expressed as mean ± SD, *n* = 3 (A, C, and D).

From the growth analysis of the single mutants, it was clear that acetate activation in *H. mediterranei* was catalyzed by multiple acetate activation enzymes. Hence, all the candidate AMP-ACS genes were knocked out one by one based on DF50ΔEPS, resulting in Δ2 (Δ1643 Δ5131), Δ3 (Δ2Δ2150), Δ4 (Δ3Δ1242), Δ5 (Δ4Δ0870), and Δ6 (Δ5Δ5129) mutants. Finally, HFX_0998 was also knocked out in Δ6, resulting in the Δ7 mutant. On analyzing their growth on 0.12 M acetate by spot assay, no difference was observed compared with that of DF50ΔEPS (Fig. S4A). Subsequently, quantitative growth of Δ7 in the liquid medium was analyzed, and the mutant showed a trivial growth reduction compared to DF50ΔEPS (Fig. 3A). Thus, Δ7 retained most of its ability to grow on acetate, indicating the presence of more acetate activation genes in *H. mediterranei*.

To screen more candidate genes involved in the acetate activation of *H. mediterranei*, the transcriptome of Δ7 was analyzed when acetate and glucose were used as the sole carbon sources for cell growth. The volcano plot illustrated the distribution of differentially expressed genes (DEGs) in the Δ7 (Fig. 3B). Overall, the expressions of 1,542 genes were significantly affected (log_2_(fold change) >0) in acetate compared to glucose. Among them, 815 genes were upregulated and 727 genes were downregulated. In the study by Pettinato *et al*. (8), it has been reported that succinyl-CoA:acetate CoA-transferase in few acetate-oxidizing sulfate reducers catalyzed acetate activation to acetyl-CoA during the conversion of succinyl-CoA to succinate. Thus, based on the annotation of CoA transferase or CoA synthetase in addition to their transcript level, five candidate genes, namely, HFX_1451 (medium-chain-fatty-acid-CoA synthetase), HFX_4020 (long-chain acyl-CoA synthetase), HFX_1837 (medium-chain acyl-CoA synthetase), HFX_1860 (CoA transferase), and HFX_5190 (putative acyl-CoA transferase), were selected with their respective log_2_ (fold change) values corresponding to 1.27, 0.45, 0.69, 3.02, and 0.31 (Fig. 3C). The proteins encoded by HFX_1451, HFX_4020, and HFX_1837 showed ~23 to 34% homology with the preliminarily screened putative AMP-ACS (HFX_0870, HFX_1242, HFX_1643, HFX_2150, HFX_5129, and HFX_5131) and ~25 to 63% homology with the functional ACS of *H. volcanii* (Table 2; Table S3). Thus, assuming that they might be functional in acetate activation, each of the three genes (HFX_1451, HFX_4020, and HFX_1837) were knocked out in Δ7, one by one. However, the resulting mutants, Δ8 (Δ7Δ1451), Δ9 (Δ8Δ4020), and Δ10 (Δ9Δ1837), showed no growth reduction on acetate compared to DF50ΔEPS and Δ7 in the spot assay (Fig. S4B). The CoA transferase encoded by HFX_1860 exhibited homology (28.33%) only with HFX_1242, whereas the HFX_5190-encoded protein showed no homology with the six putative AMP-ACS. Hence, just to check their possibility in acetate activation, they were individually deleted in Δ10, thus resulting in Δ10Δ1860 and Δ10Δ5190. Growth of these two mutants in acetate-containing liquid medium was comparable to that of Δ10 (Fig. S4C). Finally, a gene annotated as medium-chain acyl-CoA synthetase (HFX_6342) was screened. Although its mRNA expression was not induced by acetate in the Δ7 (Fig. 3C), its encoded protein showed high homology (89.78% and 68.14% identity) with the functional ACS9 and ACS2 of *H. volcanii*, respectively. On deleting HFX_6342 in Δ10, the resulting mutant Δ11 completely lost its ability to grow on acetate (Fig. 3D). Taken together, thirteen possible candidate genes for acetate activation were screened, and finally the Δ11 mutant with no capability to grow on acetate was constructed, *via* gradual deletion of eleven genes.

### Identification of the functional ACS by gene complementation

**Fig 4:**
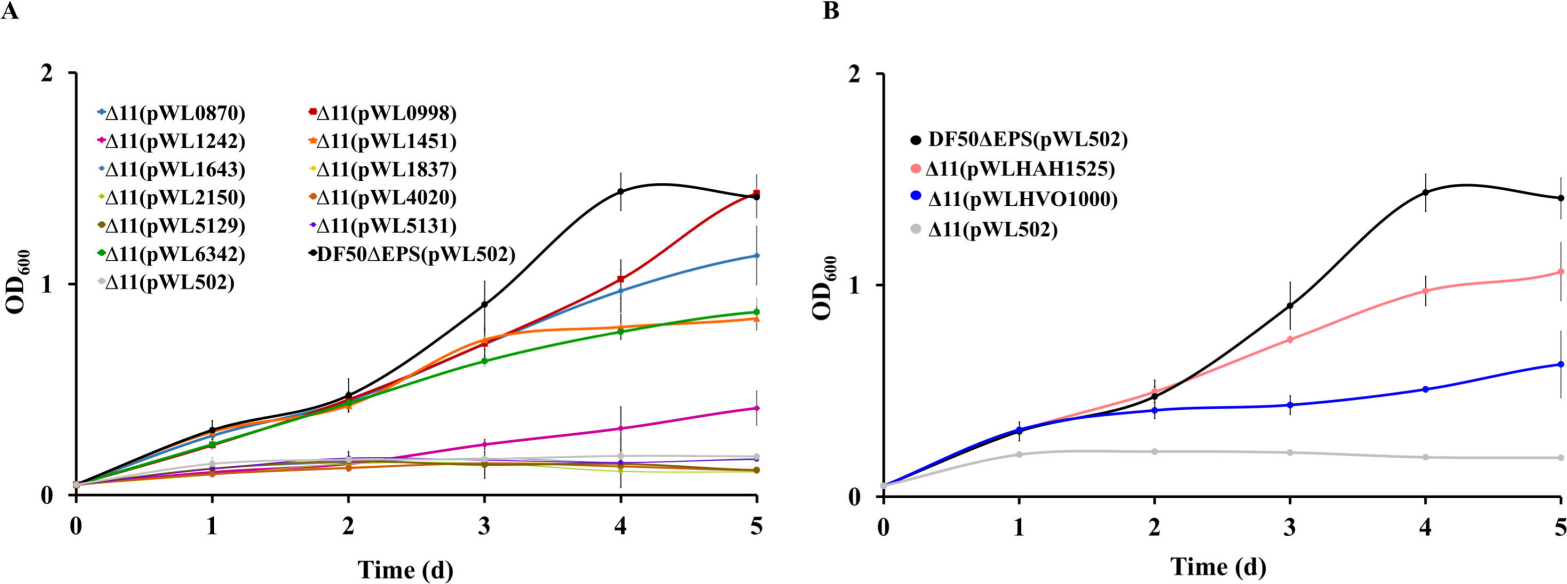
Complementation of ADP-ACS in Δ11 restored acetate activation. (**A**) Growth analysis of Δ11 complementation strains on 0.12 M acetate. Red, blue, green, orange, and purple lines indicate growth of Δ11 complemented with HFX_0998, HFX_0870, HFX_6342, HFX_1451, and HFX_1242, respectively. (**B**) Growth analysis of Δ11 with heterologous expression of ADP-ACS genes (HVO_1000 and HAH_1525) from *H. volcanii* and *H. hispanica* on 0.12 M acetate. Blue and pink lines represent the expression of HVO_1000 and HAH_1525 in Δ11, respectively. Δ11 (pWL502) and DF50ΔEPS (pWL502) represent negative (gray line) and positive control (black line), respectively. Data are expressed as mean ± SD, *n* = 3.

Given that Δ11 was unable to grow on acetate, it was necessary to identify the functional acetate activation genes in *H. mediterranei*. To realize this, each of the deleted genes was individually expressed *via* plasmid pWL502 in Δ11, under the control of their native promoters, and thereafter its ability to catalyze acetate activation was studied. In total, eleven expression plasmids were constructed (Table S1). Cell growth of all the complementation strains in the liquid medium containing 0.12 M acetate was evaluated (Fig. 4A). As expected, Δ11(pWL502) was unable to grow on acetate. However, complementation of HFX_0870, HFX_1242, HFX_1451, HFX_6342, and HFX_0998 enabled Δ11 to grow on acetate. At Day 5, the OD_600_ of DF50ΔEPS(pWL502) was 1.42 and that of Δ11(pWL0870), Δ11(pWL1242), Δ11(pWL1451), Δ11(pWL6342), and Δ11(pWL0998) were 1.13, 0.41, 0.84, 0.87, and 1.42, respectively. On the contrary, Δ11(pWL1643), Δ11(pWL1837), Δ11(pWL2150), Δ11(pWL4020), Δ11(pWL5129), and Δ11(pWL5131) were unable to grow. It was evident that the ability of HFX_0998 to catalyze acetate activation in Δ11 was highest, followed by that of HFX_0870. The acetate activation ability of HFX_1451 and HFX_6342 was almost comparable, and HFX_1242 showed the lowest ability. Notably, for the first time, the acetate activation activity of haloarchaeal ADP-ACS was observed in the plasmid-based expression system, and thus it was necessary to verify if the function was widespread in haloarchaea. The ADP-ACS encoding genes from other haloarchaea, *H. volcanii* (HVO_1000) and *H. hispanica* (HAH_1525), were expressed in Δ11, driven by their native promoter, and their growth on acetate was analyzed. Heterologous expression of the HVO_1000 or HAH_1525 enabled Δ11 to grow on acetate (Fig. 4B). At Day 5, the respective OD_600_ of DF50ΔEPS(pWL502), Δ11(pWLHVO1000), and Δ11(pWLHAH1525) was 1.41, 0.62, and 1.06. Hence, our findings indicated that acetate activation activity of ADP-ACS *via* plasmid-mediated heterologous expression is widespread among haloarchaea.

**Fig 5:**
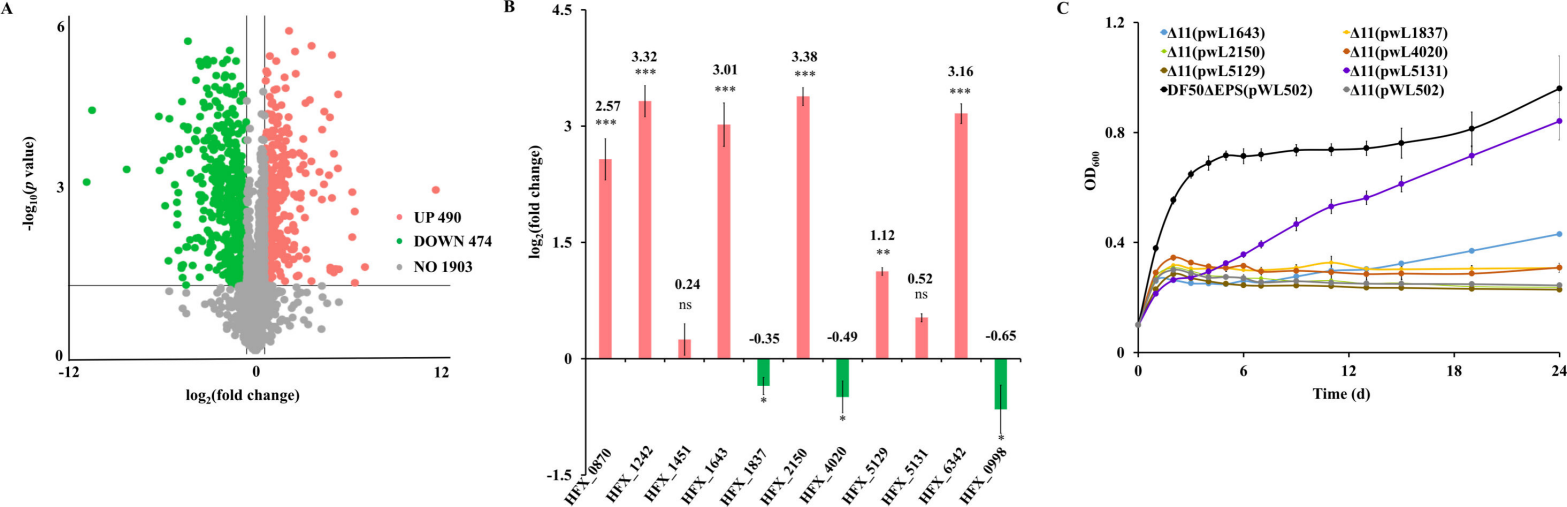
HFX_5131 and HFX_1643 involved in acetate activation at low concentration of acetate. (**A**) Volcano plot of differentially expressed proteins of DF50ΔEPS in 0.12 M acetate compared to 0.04 M glucose. Pink circles represent upregulated proteins, green circles represent downregulated proteins, and gray circles represent proteins with no change in expression. (**B**) Changes in the expression level of the eleven possible acetate activation enzymes, represented as log_2_(fold change) of expression in 0.12 M acetate compared to 0.04 M glucose. Pink columns represent upregulation of HFX_0870, HFX_1242, HFX_1451, HFX_1643, HFX_2150, HFX_5129, HFX_5131, and HFX_6342 in acetate. Green columns represent downregulation of HFX_1837, HFX_4020, and HFX_0998 in acetate. ns represents nonsignificant (*p-*value > 0.05), ** represents *p*-value < 0.01, and *** represents *p*-value < 0.005. (**C**) Growth analysis of the Δ11 complementation strain on 0.04 M acetate. Violet and blue lines represent growth of Δ11 complemented with HFX_5131 and HFX_1643, respectively. Δ11(pWL502) (gray line) and DF50ΔEPS(pWL502) (black line) represent negative and positive controls, respectively. Data are expressed as mean ± SD, *n* = 3 (**B, C**).

Among the other ten genes knocked out in Δ11, four (HFX_0870, HFX_1242, HFX_1451, and HFX_6342) were found to be functional in acetate activation. The finding was further justified using the proteome of DF50ΔEPS in acetate *vs*. glucose. As shown in the volcano plot (Fig. 5A), the expression levels of 964 proteins were significantly affected in acetate- compared to glucose-grown cells. Among them, the expressions of 490 proteins were upregulated and those of 474 proteins were downregulated. The expressions of HFX_0870, HFX_1242, HFX_1451, and HFX_6342 were upregulated in acetate (Fig. 5B), and thus, consistent with the gene complementation result. However, the protein expressions of four more genes (HFX_1643, HFX_2150, HFX_5129, and HFX_5131) were also upregulated (Fig. 5B), but its acetate activation activity was not observed in the complementation experiment. It was speculated that their acetate activation activity was possibly weak, and hence, their growth was analyzed at a low acetate concentration of 0.04 M. Interestingly, the OD_600_ of Δ11(pWL5131) and Δ11(pWL1643) showed an increasing trend from Day 3 and Day 6 onward, respectively (Fig. 5C). The increase in growth was evident when the incubation period was prolonged. At Day 15, OD_600_ of DF50ΔEPS(pWL502), Δ11(pWL5131), and Δ11(pWL1643) was 0.74, 0.61, and 0.32, respectively. At Day 24, the OD_600_ values increased to 0.96, 0.84, and 0.45, respectively. Furthermore, the acetate utilization was quantified using HPLC, and it was found that 72.5%, 50.9%, and 17.8% of acetate were utilized, respectively, by DF50ΔEPS(pWL502), Δ11(pWL5131), and Δ11(pWL1643) after 24 days of fermentation. Rest of the strains including Δ11(pWL502) did not show any increase in growth as well as acetate utilization. Thus, it was inferred that HFX_5131 and HFX_1643 were also functionally involved in acetate activation in *H. mediterranei*, although the activity of HFX_1643 was considerably weak. Taken together, seven ACS encoded by HFX_0998, HFX_0870, HFX_1451, HFX_6342, HFX_1242, HFX_5131, and HFX_1643 (in the order of decreasing acetate activation ability) were found to be functional in acetate activation when complemented in the Δ11 mutant of *H. mediterranei*.

### Genetic verification of ACS involved in acetate activation

**Fig 6:**
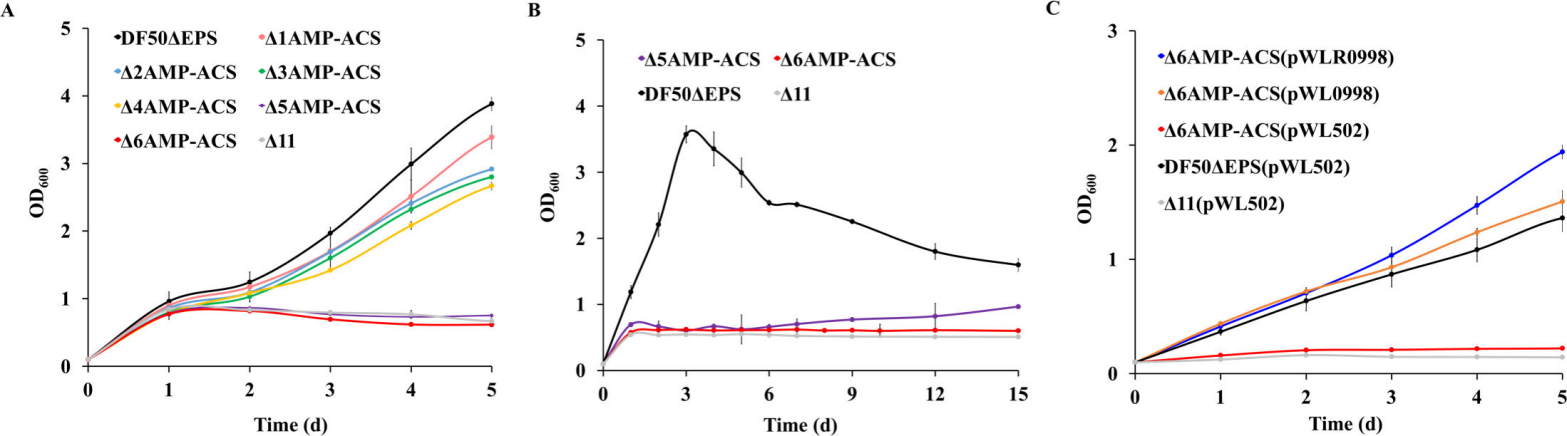
Plasmid-based overexpression of the ADP-ACS gene restored the growth of Δ6AMP-ACS on acetate. Growth of functional AMP-ACS mutants on 0.12 M (**A**) and 0.04 M (**B**) acetate. Δ1AMP-ACS represents DF50ΔEPSΔ6342 (pink line), Δ2AMP-ACS represents Δ1AMP-ACSΔ1451(blue line), Δ3AMP-ACS represents Δ2AMP-ACSΔ1242 (green line), Δ4AMP-ACS represents Δ3AMP-ACSΔ0870 (yellow line), Δ5AMP-ACS represents Δ4AMP-ACSΔ5131 (violet line), and Δ6AMP-ACS represents Δ5AMP-ACSΔ1643 (red). Δ11 (gray line) and DF50ΔEPS (black line) represent negative and positive controls, respectively. (**C**) Growth of Δ6AMP-ACS on 0.12 M acetate after HFX_0998 overexpression using the pWL502 plasmid, with the expression driven by its native promoter (orange line) or a strong promoter P*_phaR_* (dark blue line). Δ11(pWL502) (gray line) and DF50ΔEPS(pWL502) (black line) represent negative and positive controls, respectively. Data are expressed as mean ± SD, *n* = 3.

The gene complementation experiment screened seven functional acetate activation genes, which included six AMP-ACS and one ADP-ACS. To validate the finding and substantiate the role of ADP-ACS in acetate activation, each of the seven genes in DF50ΔEPS were knocked out one by one, and their growth on acetate was evaluated. The growth of mutants gradually decreased on 0.12 M acetate with deletion of each of the functional genes (Fig. 6A). Δ5AMP-ACS (DF50ΔEPS with five functional genes, namely, HFX_0870, HFX_1242, HFX_1451, HFX_6342, and HFX_5131 deleted) failed to grow on 0.12 M acetate. Keeping in mind the weak acetate activation activity of HFX_1643, as verified by the complementation experiment, it was assumed that the mutant Δ5AMP-ACS might be able to grow on a low concentration of acetate. At 0.04 M acetate, Δ5AMP-ACS started growing after Day 6 (Fig. 6B). However, when HFX_1643 was further knocked out in Δ5AMP-ACS, the resulting mutant Δ6AMP-ACS was unable to grow even on 0.04 M acetate. Hence, it proved that acetate activation in *H. mediterranei* was catalyzed by the six AMP-ACS encoded by HFX_0870, HFX_1242, HFX_1451, HFX_6342, HFX_5131, and HFX_1643, indicating that ADP-ACS encoded by HFX_0998 was unable to activate acetate in *H. mediterranei in vivo*.

Since complementation of HFX_0998 encoded ADP-ACS enabled acetate activation in Δ11, it was speculated that the low expression level of HFX_0998 driven by its native promoter on the chromosome was the main reason for its inability to catalyze acetate activation in Δ6AMP-ACS. To confirm this, HFX_0998 was overexpressed in Δ6AMP-ACS, using the pWL502 plasmid, driven by its native promoter or a strong promoter P*_phaR_*. As expected, Δ6AMP-ACS(pWL502) was unable to grow on 0.12 M acetate; however, plasmid-based overexpression of HFX_0998 in Δ6AMP-ACS restored its ability to grow on acetate (Fig. 6C). Furthermore, the overexpression strains exhibited better growth on acetate compared to DF50ΔEPS(pWL502). At Day 5, the OD_600_ of DF50ΔEPS(pWL502), Δ6AMP-ACS(pWL0998), and Δ6AMP-ACS(pWLR0998) was 1.40, 1.51, and 1.94, respectively. Hence, it was concluded that acetate activation in *H. mediterranei* was catalyzed by six AMP-ACS enzymes encoded by HFX_0870, HFX_1242, HFX_1451, HFX_6342, HFX_5131, and HFX_1643. Even though ADP-ACS possessed acetate utilization ability, owing to its low native expression level, the enzyme was unable to function in acetate activation *in vivo*. However, its plasmid-based overexpression successfully restored acetate activation in Δ6AMP-ACS.

## DISCUSSION

AMP-ACS and ADP-ACS enzymes are the two key players in acetate/acetyl-CoA interconversion in haloarchaea. *In vitro*, ADP-ACS catalyzes both the directions of this interconversion; however, *in vivo*, acetate activation is reported to be catalyzed by AMP-ACS and acetate formation is catalyzed by ADP-ACS (15, 16). In this study, thirteen possible acetate activation genes were screened by bioinformatic and transcriptome analysis in *H. mediterranei*. Subsequently, a knockout mutant, Δ6AMP-ACS, characterized by complete loss of its ability to grow on acetate was obtained *via* gradual deletion of six functional AMP-ACS genes. Intriguingly, plasmid-based overexpression of ADP-ACS in Δ6AMP-ACS restored its acetate activation and even surpassed the growth of the parent strain DF50ΔEPS on acetate. The present study showcased a systematic investigation of acetate activation in *H. mediterranei* and not only identified the functional AMP-ACS but also explored the role of ADP-ACS in the process.

With acetate becoming an alternative potential carbon source to glucose for bioproduct synthesis, much attention is being delved into exploration of the acetyl-CoA/acetate interconversion in different microbial domains. In bacteria, the key enzymes mainly involved in this interconversion are AK/PTA and AMP-ACS (30). However, several variations can be observed, as mentioned in Table S4. In haloarchaea, the AK encoding gene is absent; however, a putative PTA clustered with ADP-ACS is widespread. Glucose-specific upregulation of the PTA protein encoded by HFX_0997 was evident from our proteomic analysis, which is also in accordance with the previous findings by Kuprat *et al*. (15) in *H. volcanii* (15); however, its exact physiological function in haloarchaea remains undetermined.

Recently, the AMP-ACS encoding genes in *H. volcanii* were characterized. The genome of *H. volcanii* possessed nine ACS paralogs, out of which four were functionally involved in the acetate activation (ACS7, ACS2, ACS1, and ACS9) (9). The quadruple mutant strain lost the ability to grow on acetate. Our study screened one ADP-ACS gene (HFX_0998), two CoA transferase genes (HFX_1860 and HFX_5190), and ten putative AMP-ACS genes that were possibly involved in acetate activation in *H. mediterranei*. Among the ten genes, six were annotated as AMP-ACS (HFX_0870, HFX_1242, HFX_1643, HFX_2150, HFX_5129, and HFX_5131), three were annotated as medium-chain-acyl-CoA synthetase (HFX_1451, HFX_1837, and HFX_6342), and one was annotated as long-chain acyl-CoA synthetase (HFX_4020). Simultaneous deletion of the ten AMP-ACS and one ADP-ACS genes led to a mutant (Δ11) whose cell growth on acetate was completely abolished. The sequence alignment showed that the ten candidate AMP-ACS from *H. mediterranei* exhibited high identity with the AMP-ACS from other archaeal and bacterial species. The candidate AMP-ACS shared several conserved regions, including the four characteristic features of AMP-forming proteins (A3, A5, A8, and A10) (3, 27, 31) (Fig. S5). Region A3 and A10, characterized by the SG-rich loop and GK dipeptide, respectively, were assumed to be involved in the first half of the acetate activation reaction (acetate +ATP → acetyl-AMP +PP_i_). Moreover, the highly conserved K residue (A10 region) is known to be the site for acetylation/deacetylation regulation in AMP-forming proteins, playing a key role in the formation of the acetyl-AMP intermediate (3, 27). The regions A5 and A8, characterized by TE dipeptide and sequence DX6GXR, where G, is invariant, respectively, are assumed to be involved in substrate (acetyl-AMP and CoA) binding in the second half of the reaction (acetyl-AMP +CoA → acetyl-CoA +AMP). Furthermore, as summarized in Table S5, the ten candidate AMP-ACS also possessed AMP-binding sites similar to the AMP-forming acyl-CoA synthetases from other archaeal and bacterial species (27). The ten AMP-ACS candidates of *H. mediterranei*, AMP-ACS from *H. volcanii*, *H. marismortui*, *S. enterica* LT2 ACS, and *P. aerophilum* IM2; medium chain-acyl-CoA synthetase from *Methanosarcina acetivorans* and *Escherichia coli*; and long chain-acyl-CoA synthetase from *Mycobacterium tuberculosis* H37R conserved G in Site 1; T and E in Site 2 (belonged to the conserved A5 region); and R (and G) in Site 3, suggesting that these residues may be essential for AMP binding. However, future experimental investigations might be needed to confirm the role of these highly conserved residues in AMP-binding.

Based on genetic characterization, six genes were finally found to be functional in acetate activation in *H. mediterranei*, which included four AMP-ACS (HFX_0870, HFX_1242, HFX_1643, and HFX_5131) and two medium-chain-acyl-CoA synthetase (HFX_1451 and HFX_6342). Although the substrate specificity of HFX_1451 and HFX_6342 has not been fully determined, we tested its ability to activate acetate. Hence, they have been considered as AMP-ACS in our study. As shown in Table S3, the functional AMP-ACS of *H. mediterranei*, HFX_0870, HFX_5131, and HFX_6342 showed high homology with the functional ACS proteins, HVO_0894, HVO_A0156, and HVO_A0551 of *H. volcanii*. However, respective homologs of the other three functional AMP-ACS, HFX_1242, HFX_1451, and HFX_1643 in *H. volcanii* (namely, HVO_1236, HVO_1374, and HVO_1585) were nonfunctional in acetate activation. Meanwhile, in *H. mediterranei*, HFX_5129, HFX_5131, and HFX_5133 formed a cluster, which was highly homologous to the *H. volcanii* counterpart (Fig. S1A). HFX_5131 and HVO_A0156 (ACS7) shared 88.86% identity, and both were functional in acetate activation. Thus, it might be predicted that HFX_5133, which showed 95.6% identity with ActP of *H. volcanii*, was a candidate acetate transporter in *H. mediterranei*.

Taken together, the present study has unraveled the functional acetate activation genes in *H. mediterranei*. Additionally, the function of haloarchaeal ADP-ACS in acetate activation in the plasmid-based overexpression system has been explored for the first time. The present findings would expand our understanding of acetate metabolism in archaea, paving the way for haloarchaeal genetic engineering for enhanced acetate utilization, leading to biosynthesis of high value products.

## SUPPLEMENTAL MATERIAL

Supplemental material aem.01843-24-s0001.docx Supplemental methods, Tables S1 to S5, and Fig. S1 to S5.

ASM does not own the copyrights to Supplemental Material that may be linked to, or accessed through, an article. The authors have granted ASM a non-exclusive, world-wide license to publish the Supplemental Material files. Please contact the corresponding author directly for reuse.
