# Supplementary material for: Genetic identification of acetyl-CoA synthetases involved in acetate activation in Haloferax mediterranei
Source: Appl Environ Microbiol. 2024 Dec 31;91(1):e01843-24. doi: 10.1128/aem.01843-24 (PMC11784441; doi:10.1128/aem.01843-24)
Supplement: Supplemental material — Supplemental methods, Tables S1 to S5, and Fig. S1 to S5. [file aem.01843-24-s0001.docx]

**Supplementary materials**

**Genetic identification of acetyl-CoA synthetases involved in acetate activation in *Haloferax mediterranei***

Ruchira Mitra^a,b,#^, Yang Xu^a,c,#^, Lin Lin ^a,c,#^, Jing Guo^a,c^, Tong Xu^a^, Mengkai Zhou^a^, Feng Guo^a,d^, Hao Li^a,e^, Hua Xiang^a,c,*^, Jing Han^a,c,*^

### Affiliations

^a^ State Key Laboratory of Microbial Resources, Institute of Microbiology, Chinese Academy of Sciences, Beijing 100101, People’s Republic of China

^b^ International College, University of Chinese Academy of Sciences, Beijing, 100049, People’s Republic of China

^c^ College of Life Science, University of Chinese Academy of Sciences, 100049, Beijing, People’s Republic of China

^d^ College of Life Science, Yunnan University, 650504, Kunming, People’s Republic of China

^e^ College of Life Science, Shandong Normal University, 250014, Jinan, People’s Republic of China

^#^R.M., Y. X., and L. L. contributed equally to this work.

^*^Corresponding authors: Hua Xiang (xiangh@im.ac.cn) and Jing Han ([hanjing@im.ac.cn](mailto:hanjing@im.ac.cn)).

**Running title**: Acetate activation in *Haloferax mediterranei*

**Materials and Method**

**Cell growth determination by serial dilution-spotting assay**

The cell growth of *H. mediterranei* DF50ΔEPS and its gene deletion mutants was qualitatively determined by serial dilution-spotting assay performed on agar plates. Briefly, the *H. mediterranei* strains were grown in AS-168U liquid medium until exponential phase. Next, cell density of the strains was adjusted to be equal and serial dilutions up to 10^7^ for each strain was prepared using AS-168U medium. Then, 5 μL of each dilution was spotted onto the agar plates of fermentation medium. Photographs were taken after an incubation period of 5 days at 42ºC.

**Protein expression and purification**

The plasmids and primers used for target protein expression in *H. volcanii* H1424 are listed in Table S1 and S2. The plasmids for protein expression in *H. volcanii* H1424 were constructed based on the expression plasmid pTA06 (1). *H. volcanii* H1424 was cultivated in Hv-YPC medium at 42°C for 72 h and then cells were harvested for ultrasonication. His_6_-tagged target protein in the supernatant was purified by AKTA purifier UPC10 (GE, USA) with a HisTrap high performance (HP) column (GE, USA) according to the manufacturer’s instruction. The elution buffer used for protein purification contained 20 mM Tris-HCl, 2 M NaCl (pH 8.0) and 200 mM imidazole. Protein was analyzed by SDS-PAGE and their subsequent purity was determined by using ImageJ software. Purified protein was further identified by MALDI-TOF/TOF MS (ABI 4700; Bruker, USA). Protein concentration was determined by bicinchoninic acid (BCA) protein assay (Sangon Biotech, Shanghai) according to the manufacturer’s instruction.

**Enzyme assays**

The enzyme activity of the ADP-forming acetyl-CoA synthetase in the reaction, acetyl-CoA + ADP + P_i_ $\Leftrightarrow$ acetate + ATP + CoA, was measured in both the directions under oxic conditions at 37ºC using two different biochemical assay systems. For determination of its enzymatic activity in acetate formation, P_i_- and ADP-dependent CoA release from acetyl-CoA was measured. By using 5, 5’-dithiobis (2-nitrobenzoic acid) (or DTNB) (2), the formation of thiophenolate anion was measured after 10 min of reaction at 412 nm (Ɛ_412_=13.6 mM^-1^ cm^-1^). The assay system consisted of Tris–HCl (pH 7.5, 100 mM), KCl (3 M), MgCl_2_ (30 mM), DTNB (0.1 mM), acetyl-CoA (1.5 mM), ADP (2 mM) and KH_2_PO_4_ (5 mM). Using the equation, U = (ΔOD_412_/Δt) ·(V_total_/Ɛ·L), the enzyme activity was calculated. For the reverse direction, the activity of ADP-forming acetyl-CoA synthetase in acetate activation was determined by coupling the CoA- and acetate-dependent ADP formation from ATP with NADH oxidation to NAD^+^, catalyzed by pyruvate kinase and lactate dehydrogenase (3). This assay system consisted of Tris–HCl (pH 7.5, 100 mM), KCl (3 M), MgCl_2_ (30 mM), sodium acetate (10 mM), ATP (2 mM), CoA (1 mM), phosphoenolpyruvate (2.5 mM), NADH (0.3 mM), lactate dehydrogenase (6 U), and pyruvate kinase (4 U). Absorbance was recorded at 340 nm and enzyme activity was calculated from the standard curve of NADH. Three replicates were set for each group.

**Tables**

**Table S1** Strains and plasmids used in this study.

| **Strains or plasmid** | **Relevant characteristics** | **Source or reference** |
| --- | --- | --- |
| **Strains** |  |  |
| *Escherichia coli* JM109 | *recA1 supE44 endA1 hsdR17 gyrA96 relA1 thi* | (4) |
| *E. coli* JM110 | *dam dcm* mutant of *E. coli* JM109 | (4) |
| *Haloferax mediterranei* strains | | |
| DF50ΔEPS | *pyrF* and EPS synthesis gene cluster deletion mutant of *H. mediterranei* ATCC33500 | (5) |
| Δ0870 | HFX_0870 deletion mutant of DF50ΔEPS | This study |
| Δ0997 | HFX_0997 deletion mutant of DF50ΔEPS | This study |
| Δ0998 | HFX_0998 deletion mutant of DF50ΔEPS | This study |
| Δ1242 | HFX_1242 deletion mutant of DF50ΔEPS | This study |
| Δ1643 | HFX_1643 deletion mutant of DF50ΔEPS | This study |
| Δ2150 | HFX_2150 deletion mutant of DF50ΔEPS | This study |
| Δ5129 | HFX_5129 deletion mutant of DF50ΔEPS | This study |
| Δ5131 | HFX_5131 deletion mutant of DF50ΔEPS | This study |
| Δ2 | HFX_1643 and HFX_5131 deletion mutant of DF50ΔEPS | This study |
| Δ3 | HFX_1643, HFX_5131 and HFX_2150 deletion mutant of DF50ΔEPS | This study |
| Δ4 | HFX_1643, HFX_5131, HFX_2150, and HFX_1242 deletion mutant of DF50ΔEPS | This study |
| Δ5 | HFX_1643, HFX_5131, HFX_2150, HFX_1242, and HFX_0870 deletion mutant of DF50ΔEPS | This study |
| Δ6 | HFX_1643, HFX_5131, HFX_2150, HFX_1242, HFX_0870, and HFX_5129 deletion mutant of DF50ΔEPS | This study |
| Δ7 | HFX_1643, HFX_5131, HFX_2150, HFX_1242, HFX_0870, HFX_5129, and HFX_0998 deletion mutant of DF50ΔEPS | This study |
| Δ8 | HFX_1643, HFX_5131, HFX_2150, HFX_1242, HFX_0870, HFX_5129, HFX_0998, and HFX_1451 deletion mutant of DF50ΔEPS | This study |
| Δ9 | HFX_1643, HFX_5131, HFX_2150, HFX_1242, HFX_0870, HFX_5129, HFX_0998, HFX_1451, and HFX_4020 deletion mutant of DF50ΔEPS | This study |
| Δ10 | HFX_1643, HFX_5131, HFX_2150, HFX_1242, HFX_0870, HFX_5129, HFX_0998, HFX_1451, HFX_4020, and HFX_1837 deletion mutant of DF50ΔEPS | This study |
| Δ10Δ1860 | HFX_1643, HFX_5131, HFX_2150, HFX_1242, HFX_0870, HFX_5129, HFX_0998, HFX_1451, HFX_4020, HFX_1837, and HFX_1860 deletion mutant of DF50ΔEPS | This study |
| Δ10Δ5190 | HFX_1643, HFX_5131, HFX_2150, HFX_1242, HFX_0870, HFX_5129, HFX_0998, HFX_1451, HFX_4020, HFX_1837, and HFX_5190 deletion mutant of DF50ΔEPS | This study |
| Δ11 | HFX_1643, HFX_5131, HFX_2150, HFX_1242, HFX_0870, HFX_5129, HFX_0998, HFX_1451, HFX_4020, HFX_1837, and HFX_6342 deletion mutant of DF50ΔEPS | This study |
| Δ1AMP-ACS | HFX_6342 deletion mutant of DF50ΔEPS | This study |
| Δ2AMP-ACS | HFX_6342 and HFX_1451 deletion mutant of DF50ΔEPS | This study |
| Δ3AMP-ACS | HFX_6342, HFX_1451, and HFX_1242 deletion mutant of DF50ΔEPS | This study |
| Δ4AMP-ACS | HFX_6342, HFX_1451, HFX_1242 and HFX_0870 deletion mutant of DF50ΔEPS | This study |
| Δ5AMP-ACS | HFX_6342, HFX_1451, HFX_1242, HFX_0870, and HFX_5131 deletion mutant of DF50ΔEPS | This study |
| Δ6AMP-ACS | HFX_6342, HFX_1451, HFX_1242, HFX_0870, HFX_5131, and HFX_1643 deletion mutant of DF50ΔEPS | This study |
| *Haloferax volcanii* H1424 | Δ*pyrE2* Δ*hdrB pitANph* Δ*mrr cdc48d-Ct* | (6) |
| **Plasmids** | | |
| pHFX | 4.0 kb; suicide vector containing *pyrF* and its native promoter, Amp^r^ | (7) |
| pHFX-B60 | 5.0 kb; modified pHFX containing *crtB* and P*_phaR_* | (4) |
| pHFX-0997 | 5.0 kb; modified pHFX for HFX_0997 knockout | This study |
| pHFX-0998 | 5.0 kb; modified pHFX for HFX_0998 knockout | This study |
| pHFX-5131 | 5.0 kb; modified pHFX for HFX_5131 knockout | This study |
| pHFX-B60-0870 | 6.0 kb; modified pHFX-B60 for HFX_0870 knockout | This study |
| pHFX-B60-1242 | 6.0 kb; modified pHFX-B60 for HFX_1242 knockout | This study |
| pHFX-B60-1643 | 6.0 kb; modified pHFX-B60 for HFX_1643 knockout | This study |
| pHFX-B60-2150 | 6.0 kb; modified pHFX-B60 for HFX_2150 knockout | This study |
| pHFX-B60-5129 | 6.0 kb; modified pHFX-B60 for HFX_5129 knockout | This study |
| pHFX-B60-1451 | 5.8 kb; modified pHFX-B60 for HFX_1451 knockout | This study |
| pHFX-B60-4020 | 5.8 kb; modified pHFX-B60 for HFX_4020 knockout | This study |
| pHFX-B60-1837 | 5.8 kb; modified pHFX-B60 for HFX_1837 knockout | This study |
| pHFX-B60-1860 | 5.8 kb; modified pHFX-B60 for HFX_1860 knockout | This study |
| pHFX-B60-5190 | 5.8 kb; modified pHFX-B60 for HFX_5190 knockout | This study |
| pHFX-B60-6342 | 5.8 kb; modified pHFX-B60 for HFX_6342 knockout | This study |
| pWL502 | 7.8 kb; shuttle vector with *pyrF* marker, Amp^r^ | (1) |
| pWLR502 | 7.8 kb; modified pWL502 with strong P*_phaR_* promoter | (8) |
| pWL0870 | 10.3 kb; modified pWL502 expressing HFX_0870 driven by its native promoter | This study |
| pWL0998 | 9.9 kb; modified pWL502 expressing HFX_0998 driven by its native promoter | This study |
| pWL1242 | 9.9 kb; modified pWL502 expressing HFX_1242 driven by its native promoter | This study |
| pWL1451 | 9.5 kb; modified pWL502 expressing HFX_1451 driven by its native promoter | This study |
| pWL1643 | 10.1 kb; modified pWL502 expressing HFX_1643 driven by its native promoter | This study |
| pWL1837 | 9.6 kb; modified pWL502 expressing HFX_1837 driven by its native promoter | This study |
| pWL2150 | 9.8 kb; modified pWL502 expressing HFX_2150 driven by its native promoter | This study |
| pWL4020 | 9.7 kb; modified pWL502 expressing HFX_4020 driven by its native promoter | This study |
| pWL5129 | 10.1 kb; modified pWL502 expressing HFX_5129 driven by its native promoter | This study |
| pWL5131 | 10.1 kb; modified pWL502 expressing HFX_5131 driven by its native promoter | This study |
| pWL6342 | 9.7 kb; modified pWL502 expressing HFX_6342 driven by its native promoter | This study |
| pWLHAH1525 | 10.0 kb; modified pWL502 expressing HAH_1525 driven by its native promoter | This study |
| pWLHVO1000 | 10.1 kb; modified pWL502 expressing HVO_1000 driven by its native promoter | This study |
| pWLR0998 | 9.9 kb; modified pWL502 expressing HFX_0998 driven by P*_phaR_* promoter | This study |
| pTA06 | 8.0 kb; expression vector with N-terminal His_6_ tag and promoter P*_phaR_* | (6) |
| pTA06-0998 | 10.1 kb; modified pTA06 expressing HFX_0998 driven by P*_phaR_* promoter | This study |

**Table S2** Primers used in this study.

| **Primers** | **Sequence (5'→3')** | **Usage** |
| --- | --- | --- |
| HFX_0997-F1 | ATAGGATCCCGCCGCAGGGAGCTATCA | Amplification of homologous arm sequences of HFX_0997 for insertion into pHFX to construct pHFX-0997 |
| HFX_0997-R1 | GCGCCATGGTATTGAGTGGTTGGTGTC |  |
| HFX_0997-F2 | GCGCCATGGCGTCGGTGTGTGACCAAC |  |
| HFX_0997-R2 | ATAGGTACCGCGACATGGGCGAAGACG |  |
| HFX_0998-F1  HFX_0998-R1  HFX_0998-F2  HFX_0998-R2 | CGCGGATCCGGCCGAAATCAGAGAACC  TGTCTGAAGTGTTTAACCCATTACCAGTTGAT  ATCAACTGGTAATGGGTTAAACACTTCAGACA  CGGGGTACCCGCTCGAATGCTGGTAGT | Amplification of homologous arm sequences of HFX_0998 for insertion into pHFX to construct pHFX-0998 |
| HFX_5131-F1  HFX_5131-R1  HFX_5131-F2  HFX_5131-R2 | CGCGGATCCCGCGTACCCCGACACCGA  CGGTAGTTGGTGTTCAATGTCATGCTAACGAG  CTCGTTAGCATGACATTGAACACCAACTACCG  CGGGGTACCCCCGTCAACGTCATTCGA | Amplification of homologous arm sequences of HFX_5131 for insertion into pHFX to construct pHFX-5131 |
| HFX_0870-F1 | GCGTGGCGTGGATGAGATATCGAGCTCGTGATTCGCTTTGAGACGGC | Amplification of homologous arm sequences of HFX_0870 for insertion into pHFX-B60 to construct pHFX-B60-0870 |
| HFX_0870-R1 | CAACCGTGCCTCAAGTTCAC |  |
| HFX_0870-F2 | CTTGAGGCACGGTTGGACACCGAGTAGCCACCTG |  |
| HFX_0870-R2 | TATAGGGAGAAGCTTGCATGCCGTCGAGTCTAGCGGAGAAC |  |
| HFX_1242-F1 | GCGTGGCGTGGATGAGATATCGAGCTCTGGGTGTTTTCGGGCTACTG | Amplification of homologous arm sequences of HFX_1242 for insertion into pHFX-B60 to construct pHFX-B60-1242 |
| HFX_1242-R1 | TGGCTCGACAGACAGAGAGG |  |
| HFX_1242-F2 | CTGTCTGTCGAGCCAAGCGCGAATAGGTCAGAAGA |  |
| HFX_1242-R2 | TATAGGGAGAAGCTTGCATGCCGACTTGTCCCACATCCACT |  |
| HFX_1643-F1 | GCGTGGCGTGGATGAGATATCGAGCTCGACCTGTTCGAGTCGGCTTC | Amplification of homologous arm sequences of HFX_1643 for insertion into pHFX-B60 to construct pHFX-B60-1643 |
| HFX_1643-R1 | CGTCCATGAATCGACGTACTG |  |
| HFX_1643-F2 | GTCGATTCATGGACGCGTCCTGAAGAGACGACTGC |  |
| HFX_1643-R2 | TATAGGGAGAAGCTTGCATGCGCGTATTTACGGCTTCTCGC |  |
| HFX_2150-F1 | GCGTGGCGTGGATGAGATATCGAGCTCACCCATTCTGTGAAGCCGTT | Amplification of homologous arm sequences of HFX_2150 for insertion into pHFX-B60 to construct pHFX-B60-2150 |
| HFX_2150-R1 | CGTAGTCGCCGAGATTGTGT |  |
| HFX_2150-F2 | ATCTCGGCGACTACGCGGCTGAAGAGAACTGAG |  |
| HFX_2150-R2 | TATAGGGAGAAGCTTGCATGCGGTCGGAGGGATTACACT |  |
| HFX_5129-F1 | GCGTGGCGTGGATGAGATATCGAGCTCACATCGCACTTCTCCCTTCG | Amplification of homologous arm sequences of HFX_5129 for insertion into pHFX-B60 to construct pHFX-B60-5129 |
| HFX_5129-R1 | TGCACAAATCCAATCGCCCT |  |
| HFX_5129-F2 | GATTGGATTTGTGCACCATCGTTCGGGACAACTG |  |
| HFX_5129-R2 | TATAGGGAGAAGCTTGCATGCGCCCTCGTGACTGGTCTTT |  |
| HFX_1451-F1 | GTGGATGAGATATCGAGCTCGACGTTTCAGACAGC | Amplification of homologous arm sequences of HFX_1451 for insertion into pHFX-B60 to construct pHFX-B60-1451 |
| HFX_1451-R1 | GTGAGTGGCGTGAAAACGTTCCCCTTGTGGTAAAA |  |
| HFX_1451-F2 | CCACAAGGGGAACGTTTTCACGCCACTCACACTTT |  |
| HFX_1451-R2 | ATAGGGAGAAGCTTGCATGCGTCGTCTCCATGCCA |  |
| HFX_4020-F1 | GTGGATGAGATATCGAGCTCGACTGGAAGCTCCTCATGCA | Amplification of homologous arm sequences of HFX_4020 for insertion into pHFX-B60 to construct pHFX-B60-4020 |
| HFX_4020-R1 | GCCATCTCTGTCGCTCCGGGTCCAGCGGGACATCGTAGTT |  |
| HFX_4020-F2 | AACTACGATGTCCCGCTGGACCCGGAGCGACAGAGATGGC |  |
| HFX_4020-R2 | ATAGGGAGAAGCTTGCATGCCAACCACTGTTAATAGTAAT |  |
| HFX_1837-F1 | GTGGATGAGATATCGAGCTCGAGCGCCGTGAAGCGAACGT | Amplification of homologous arm sequences of HFX_1837 for insertion into pHFX-B60 to construct pHFX-B60-1837 |
| HFX_1837-R1 | CTTAGCCTTCATCGACCGACAGGTATAGCATGACAATATC |  |
| HFX_1837-F2 | GATATTGTCATGCTATACCTGTCGGTCGATGAAGGCTAAG |  |
| HFX_1837-R2 | ATAGGGAGAAGCTTGCATGCACGGGCGCACACAGGACGAG |  |
| HFX_1860-F1 | GTGGATGAGATATCGAGCTCTTATCTGGATCAACGTCGGC | Amplification of homologous arm sequences of HFX_1860 for insertion into pHFX-B60 to construct pHFX-B60-1860 |
| HFX_1860-R1 | GGAAGGTCCGGCGGAGGCGGGAGAAGTCTGGCTGACCGAG |  |
| HFX_1860-F2 | CTCGGTCAGCCAGACTTCTCCCGCCTCCGCCGGACCTTCC |  |
| HFX_1860-R2 | ATAGGGAGAAGCTTGCATGCTACTCGTGGAGTTGTGCGGG |  |
| HFX_5190-F1 | GTGGATGAGATATCGAGCTCTAATCGACCCGACCGCTCAC | Amplification of homologous arm sequences of HFX_5190 for insertion into pHFX-B60 to construct pHFX-B60-5190 |
| HFX_5190-R1 | TTACCAGTCGAAGTTCACAAGTCCCATACTCGCTGCGAGC |  |
| HFX_5190-F2 | GCTCGCAGCGAGTATGGGACTTGTGAACTTCGACTGGTAA |  |
| HFX_5190-R2 | ATAGGGAGAAGCTTGCATGCTTCGTTCCGGGCGAGGTCAT |  |
| HFX_6342-F1 | GTGGATGAGATATCGAGCTCTAGTAGTCGCTTGCGGTGTT | Amplification of homologous arm sequences of HFX_6342 for insertion into pHFX-B60 to construct pHFX-B60-6342 |
| HFX_6342-R1 | ACGCAGACAGCCGTTGAGAGTCATGATAGACCGGGCTGTC |  |
| HFX_6342-F2 | GACAGCCCGGTCTATCATGACTCTCAACGGCTGTCTGCGT |  |
| HFX_6342-R2 | ATAGGGAGAAGCTTGCATGCAATCATGACATTGGTGAACT |  |
| 0870-F | CAACAACCCCCCATGGATCCCTTTGAGACGGCTGCTAAAT | Amplification of HFX_0870 for insertion into pWL502 to construct pWL0870 |
| 0870-R | GCACACAAGAAAACGGTACCCTACTCGGTGTCGACCTGTT |  |
| 0998-F | GATCCCAAGCTTCTTCTAGAATATGTGCCGTTATT | Amplification of HFX_0998 for insertion into pWL502 to construct pWL0998 |
| 0998-R | GCACACAAGAAAACGGTACCTTAAAGTTCGTCCGG |  |
| 1242-F | CAACAACCCCCCATGGATCCTCGCCCGCCGCGTGACGGGA | Amplification of HFX_1242 for insertion into pWL502 to construct pWL1242 |
| 1242-R | GCACACAAGAAAACGGTACCCTATTCGCGCTCTTGGGACC |  |
| 1451-F | CAACAACCCCCCATGGATCCACACAGTTGGTGTGTGTTTC | Amplification of HFX_1451 for insertion into pWL502 to construct pWL1451 |
| 1451-R | GCACACAAGAAAACGGTACCTCAGTTCCCCGATGGAGCCT |  |
| 1643-F | GATCCCAAGCTTCTTCTAGAACGTAGTCGCGGGAG | Amplification of HFX_1643 for insertion into pWL502 to construct pWL1643 |
| 1643-R | GCACACAAGAAAACGGTACCTCAGGACGCCTGTTC |  |
| 1837-F | CAACAACCCCCCATGGATCCGCACGGACTGAGAGACACCG | Amplification of HFX_1837 for insertion into pWL502 to construct pWL1837 |
| 1837-R | GCACACAAGAAAACGGTACCTCATCCCTCACCGACCATCC |  |
| 2150-F | GATCCCAAGCTTCTTCTAGAACTCCGAACCAACAG | Amplification of HFX_2150 for insertion into pWL502 to construct pWL2150 |
| 2150-R | GCACACAAGAAAACGGTACCTCAGTTCTCTTCAGC |  |
| 4020-F | CAACAACCCCCCATGGATCCTACTGTCGGCTGTTAATTCT | Amplification of HFX_4020 for insertion into pWL502 to construct pWL4020 |
| 4020-R | GCACACAAGAAAACGGTACCTCACTTGGTTGCCTCGGTCT |  |
| 5129-F | CAACAACCCCCCATGGATCCACAACACAACGAATCATCAT | Amplification of HFX_5129 for insertion into pWL502 to construct pWL5129 |
| 5129-R | GCACACAAGAAAACGGTACCTCAGTTGTCCCGAACGATGG |  |
| 5131-F | GATCCCAAGCTTCTTCTAGAGCCTTTAGGGTGTGA | Amplification of HFX_5131 for insertion into pWL502 to construct pWL5131 |
| 5131-R | GCACACAAGAAAACGGTACCTCAGTCGCTCTGTGC |  |
| 6342-F | GATCCCAAGCTTCTTCTAGAACGAGAAGGGTCGGC | Amplification of HFX_6342 for insertion into pWL502 to construct pWL6342 |
| 6342-R | GCACACAAGAAAACGGTACCCTACCCCTTCCCGAC |  |
| HAH_1525-F | CAACAACCCCCCATGGATCCCGGACGCCGCTACGGGGGTG | Amplification of HAH_1525 for insertion into pWL502 to construct pWLHAH1525 |
| HAH_1525-R | GCACACAAGAAAACGGTACCTCATAGTTTCTCCTGATCGA |  |
| HVO_1000-F | CAACAACCCCCCATGGATCCAGCCCGTCGTTTTCAGGCGA | Amplification of HVO_1000 for insertion into pWL502 to construct pWLHVO1000 |
| HVO_1000-R | GCACACAAGAAAACGGTACCTTAAAGTTCGTCCGGGTCCA |  |
| 0998-PhaR-F | CCGAGTTAGGAGATGGGATCCATGGGAGAGCTATCCGAATT | Amplification of HFX_0998 for insertion into pWLR502 to construct pWLR0998 |
| 0998-PhaR-R | CGCACACAAGAAAACGGTACCTCTAGAAAAAAAAATTAAAGTTCGTCCGGGTCGACGGTGA |  |
| pWL502-F | ATGCGTTGTATTCGGGTATC | For DNA sequencing of constructed plasmids based on pWL502 |
| pWL502-R | CCTTCTCGATGCGGTCCTGA |  |
| pWLR502-F | GTCGACGAACTCTGAACC | For DNA sequencing of constructed plasmids based on pWLR502 |
| pWLR502-R | GCGACGACCGTATGTAAG |  |
| pHFX-B60-F | CCGAAGCGACGTTCTACACC | For DNA sequencing of constructed plasmids based on pHFX-B60 |
| pHFX-B60-R | AGGCACCCCAGGCTTTAC |  |
| RT-0998-F | AGTGTTACGGAGACATCGGC | Analysis of HFX_0998 transcription by qRT-PCR |
| RT-0998-R | TCATGACGCCCAGACTGTTC |  |
| RT-0870-F | TGCTGGATTGGGATGACGAC | Analysis of HFX_0870 transcription by qRT-PCR |
| RT-0870-R | CCTGATACGTGTAGGTCCGC |  |
| RT-1242-F | GAACGAACCTACGACGACCT | Analysis of HFX_1242 transcription by qRT-PCR |
| RT-1242-R | TGTCCTTGGGCTTGAGCATC |  |
| RT-1643-F | TCGAAGATGCCAACGTGACT | Analysis of HFX_1643 transcription by qRT-PCR |
| RT-1643-R | GTTGTGGGCGATGTTGAGTTC |  |
| RT-2150-F | CGACGCGAGCCGACTTCT | Analysis of HFX_2150 transcription by qRT-PCR |
| RT-2150-R | GCGCGGCATGTACGAGAC |  |
| RT-5129-F | CGAATTGACCCTCCCACGTC | Analysis of HFX_5129 transcription by qRT-PCR |
| RT-5129-R | TTCTTCGCTCCGGTTTCGAT |  |
| RT-5131-F | GGAAGAACAGGCGGAATT | Analysis of HFX_5131 transcription by qRT-PCR |
| RT-5131-R | TTGGAGGAGGCGTTTAGC |  |
| RT-7S-F | ACTAGGTCGGGCAGTTAGG | Quantification of 7S rRNA for qRT-PCR |
| RT-7S-R | CGAAGGACGAGGTTTCTACG |  |
| 0998-06-F | TTCCACCACCACCACCACCACGGTACCATGGGAGAGCTATCCGAATTG | Amplification of HFX_0998 for insertion into pTA06 to construct pTA06-0998 |
| 0998-06-R | CTCTAGAACTAGTGGATCTTAGGATCCTTAAAGTTCGTCCGGGTCG |  |

Sequences representing restriction sites are underlined.

**Table S3** Amino acid sequence homology between putative AMP-ACS of *H. mediterranei* and *H. volcanii*.

|  | **HVO_0894** | **HVO_0896** | **HVO_A0156** | **HVO_A0551** | **HVO_1236** | **HVO_1374** | **HVO_1585** | **HVO_1917** | **HVO_A0158** |
| --- | --- | --- | --- | --- | --- | --- | --- | --- | --- |
| **HFX_0870** | **93.05** | 24.96 | **85.89** | 24.46 | 29.74 | 26.84 | 37.31 | 29.73 | 64.32 |
| **HFX_1242** | 30.39 | 34.89 | 29.84 | 34.58 | **89.35** | 29.71 | 33.33 | 32.62 | 31.05 |
| **HFX_1451** | 26.99 | 35.4 | 25.64 | 35.19 | 28.21 | **90.02** | 24.91 | 25.90 | 26.08 |
| **HFX_6342** | 22.95 | 68.14 | 22.47 | **89.78** | 31.02 | 36.04 | 23.05 | 31.58 | 25.05 |
| **HFX_5131** | **87.01** | 25.54 | **88.86** | 24.63 | 30.99 | 24.91 | 39.39 | 28.65 | 66.16 |
| **HFX_1643** | 37.96 | 25.10 | 37.54 | 25.61 | 31.02 | 24.91 | **90.27** | 26.09 | 37.05 |
| **HFX_1837** | 23.08 | 63.60 | 22.74 | 59.12 | 34.36 | 35.40 | 23.73 | 26.14 | 24.12 |
| **HFX_2150** | 32.49 | 31.74 | 32.62 | 31.12 | 40.15 | 28.40 | 32.70 | 33.73 | 33.27 |
| **HFX_4020** | 24.39 | 34.41 | 25 | 35.93 | 31.68 | 30.74 | 27.84 | 28.93 | 26.92 |
| **HFX_5129** | 64.99 | 26.21 | 65.35 | 25.97 | 29.31 | 25.76 | 38.12 | 27.31 | **84.00** |

Blue colour represents functional AMP-ACS. The homology is represented in percentage (%). Homology higher than 80% is represented in red.

**Table S4** Key enzymes involved in acetate activation and acetate formation in various microorganisms.

| **Domain** | **Microorganism** | **Enzymes for acetate activation** | **Enzymes for acetate formation** | **Reference** |
| --- | --- | --- | --- | --- |
| *Bacteria* | *E. coli* | AMP-ACS | AK/PTA | (9-11) |
|  |  | AK/PTA | Pyruvate oxidase |  |
|  | *Corynebaetcterium glutamicum* | CoA transferase encoded by *cat* gene | CoA transferase  encoded by *ctfA* gene | (12,13) |
|  |  | AK/PTA | AK/PTA |  |
|  |  |  | Unknown pathway *via* acetyl-CoA |  |
|  | *Bacillus subtilis* | AMP-ACS | AK/PTA | (14,15) |
|  |  |  | Unknown pathway |  |
|  | *Syntrophus aciditrophicus* | AMP-ACS | AMP-ACS | (16) |
|  | *Cutibacterium granulosum* | SCACT/SCS | SCACT/SCS | (17) |
|  | *Snodgrassella alvi* | Modified SCACT (also named as ASCT) | Modified SCACT (also named as ASCT) | (18) |
|  | *Acetobacter aceti* | Modified SCACT | Modified SCACT | (19) |
|  | *Desulfurella acetivorans* | Modified SCACT | AK-PTA | (20) |
|  |  | AK-PTA |  |  |
| *Archaea* | *Pyrococcus furiosus* | AMP-ACS | ADP-ACS | (21) |
|  | *Methanosarcina* species | AK-PTA | AK-PTA | (22,23) |
|  | *H. volcanii* | AMP-ACS | ADP-ACS | (24,25) |
|  | *H. marismortui* | AMP-ACS | ADP-ACS | (26,27) |
|  | *H. mediterranei* | AMP-ACS | ADP-ACS | This study |

**Table S5** AMP-binding sites present in ten candidate AMP-ACS of *H. mediterranei* and AMP-forming acyl-CoA synthetases from other archaeal and bacterial species.

| **Acyl-CoA synthetase** | **Site 1** | **Site 2** | **Site 3** |
| --- | --- | --- | --- |
| HFX_0870 | VGEP | DTWWQTE | ILGRVD |
| HFX_5131 | VGEP | DTWWQTE | ILGRVD |
| HFX_5129 | VGEP | DTWWQTE | VLGRVD |
| HVO_0894 | VGEP | DTWWQTE | ILGRVD |
| HVO_A0156 | VGEP | DTWWQTE | VLGRVD |
| AMP-ACS*_hm_* | VGEP | DTWWQTE | FLGRID |
| AMP-ACS*_se_* | VGEP | DTWWQTE | ITGRVD |
| AMP-ACS*_pa_* | VGEP | DTWWQTE | IVGRVD |
| HFX_1242 | AGEP | DGYGQTE | FEGRAD |
| HFX_1643 | TGEP | N I S GGTE | LHGRAD |
| HFX_2150 | IGEP | DTYGQTE | FEGRAD |
| HFX_1451 | GGSA | HAWGMTE | IVDRAK |
| HFX_4020 | GGSG | EGYGLTE | VVDRKK |
| HFX_1837 | GGAA | QMYGLTE | IQDRKK |
| HFX_6342 | AGSA | EGYGLTE | IQDRKK |
| HVO_A0551 | AGSA | QLYGATE | IQDRKK |
| HVO_0896 | AGAA | HVYGATE | IQDRKK |
| MACS*_ma_* | AGEP | EGFGQTE | FVGRAD |
| MACS*_ec_* | GGTT | SVYGSTE | ITGRKK |
| LACS*_mt_* | SGSQ | NMYGHTE | VSGRDD |

AMP-ACS*_hm_*, AMP-ACS*_se_*, and AMP-ACS*_pa_* represent AMP-ACS from *Haloarcula marismortui*, *Salmonella enterica* LT2 ACS, and *Pyrobaculum aerophilum* IM2, respectively. MACS*_ma_*, and MACS*_ec_* represent medium-chain-acyl-CoA synthetase from *Methanosarcina acetivoran* and *Escherichia coli*, respectively. LACS*_mt_* represent long-chain- acyl-CoA synthetase from *Mycobacterium tuberculosis* H37R. NCBI accession number: HVO_0894, ADE04845.1; HVO_0896, ADE02736.1; HVO_A0156, ADE02088.1; HVO_A0551, ADE02013.1; AMP-ACS*_hm_*, AAV47932.1; AMP-ACS*_se_*, Q8ZKF6; AMP-ACS*_pa_*, NP_560315. MACS*_ma_*, NP_617808.1; MACS*_ec_*, CAK1351208.1; LACS*_mt_*, P95227.

**Figures**

**
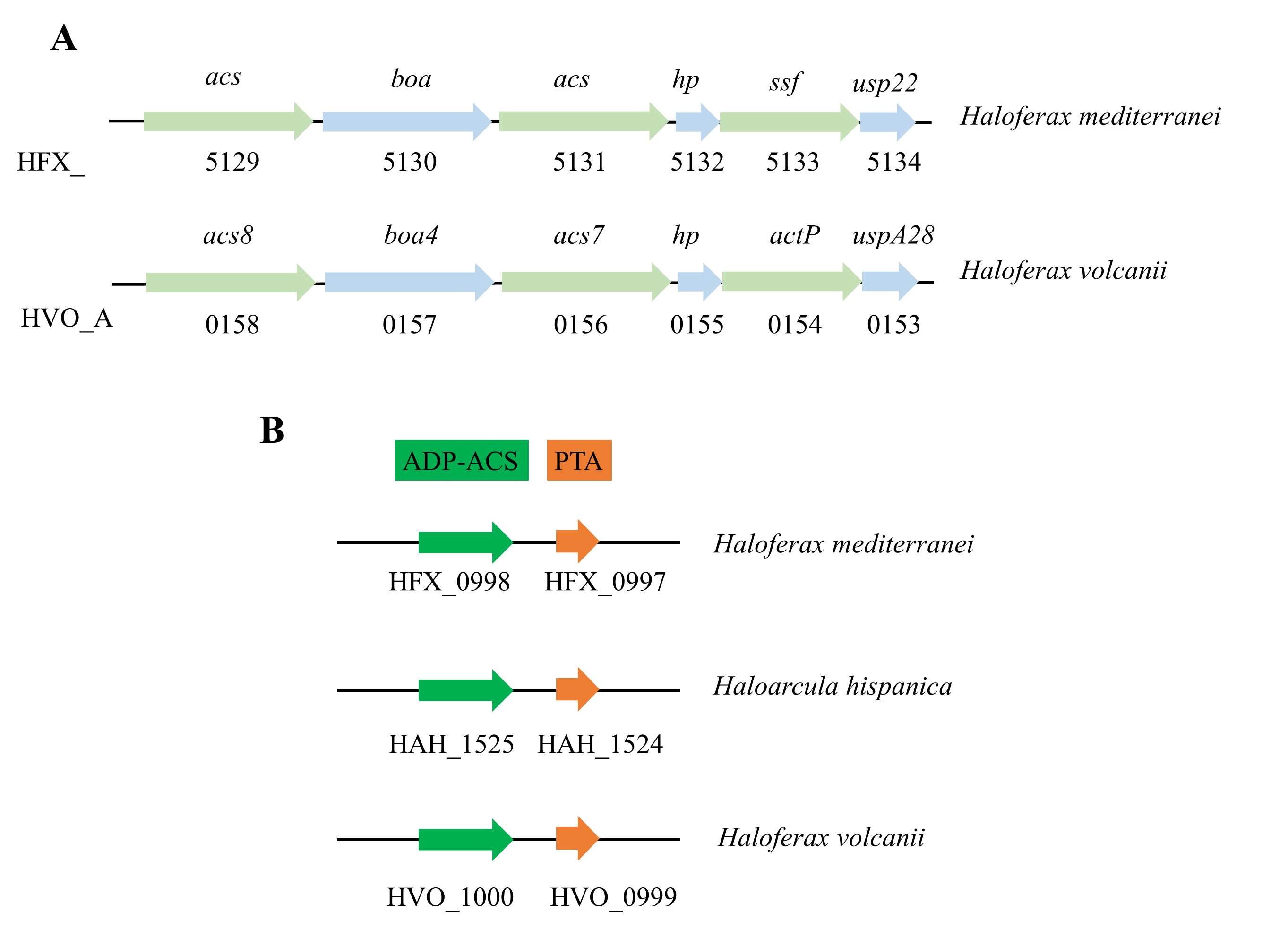
**

**Fig. S1 Genetic organization of ActP-ACS and ACS-PTA genes in haloarchaea.** (A) Genetic organization of genes encoding AMP-ACS and acetate transporter in *H. mediterranei* and *H. volcanii*. *acs*: acetyl-CoA synthetase, *boa*: bacterio-opsin activator-like protein, *hp*: hypothetical protein, *ssf*: sodium/solute symporter, *usp*: universal stress protein, *actP*: acetate transporter. (B) Genetic organization of ADP-forming acetyl-CoA synthetase (ADP-ACS) and phosphotransacetylase (PTA) in *H. mediterranei*, *H. volcanii*, and *H. hispanica*.

**
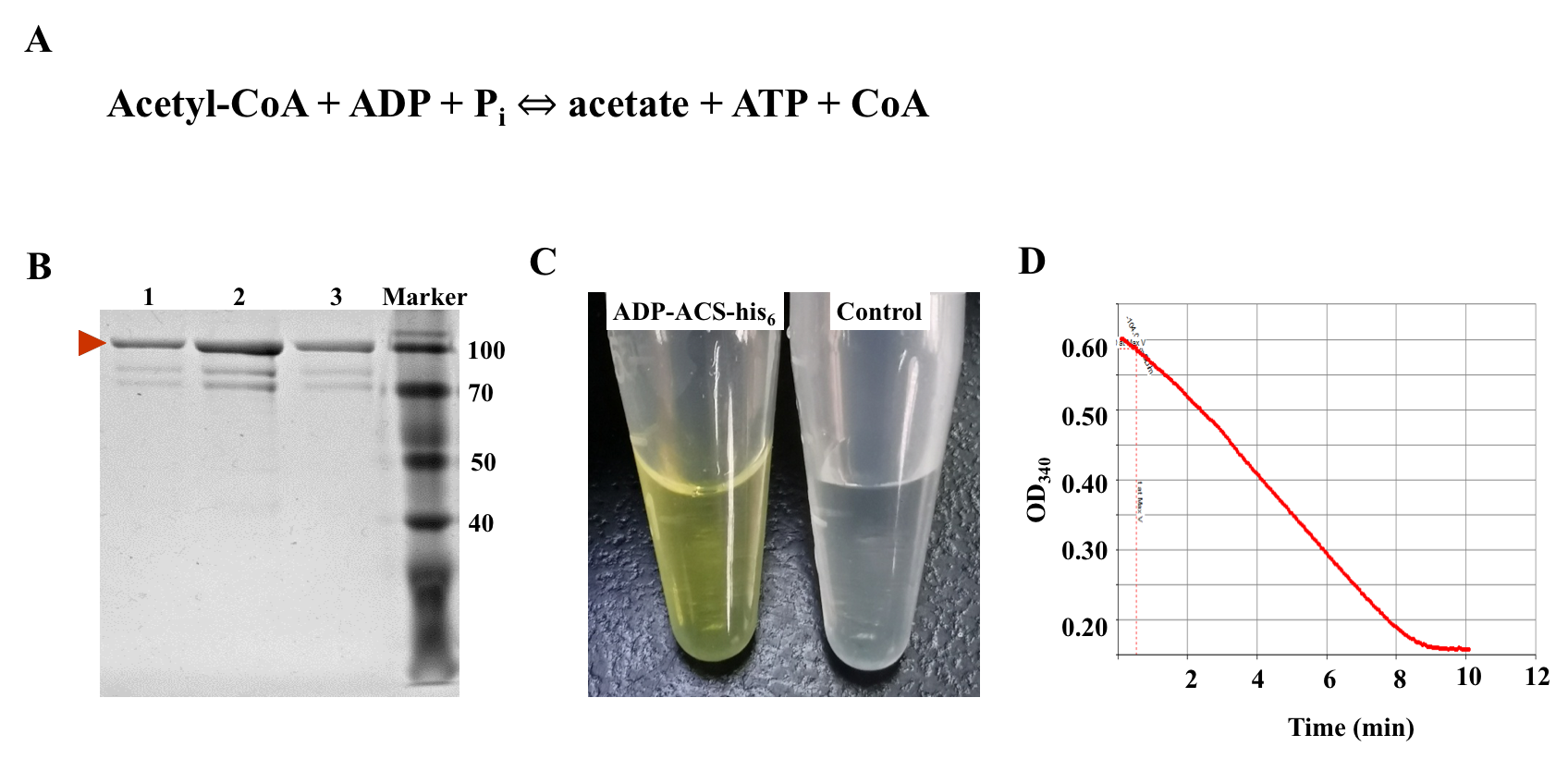
**

**Fig. S2 HFX_0998 encoded ADP-ACS catalyzed acetate/acetyl-CoA interconversion *in vitro.*** (A) Chemical reaction catalyzed by ADP-ACS. (B) SDS-PAGE analysis of purified His-tagged HFX_0998 encoded protein. The red triangle indicates the target protein ADP-ACS-His_6_. (C) Detection of CoA release from acetyl-CoA during acetate formation using DTNB method. Addition of DTNB turned colorless reaction liquid to yellow. The specific enzyme activity was calculated to be 0.27 ± 0.02 U/mg. Control reaction tube consisted of dH_2_O instead of HFX_0998 encoded protein. (D) Enzyme kinetic reaction curve for determination of acetate activation activity. ADP formation from ATP was measured at 340 nm by coupling the reaction with NADH oxidation to NAD^+^, catalyzed by pyruvate kinase and lactate dehydrogenase. The specific enzyme activity was calculated to be 0.52 ± 0.02 U/mg. Three replicates were performed and only representative data was shown (B and C).


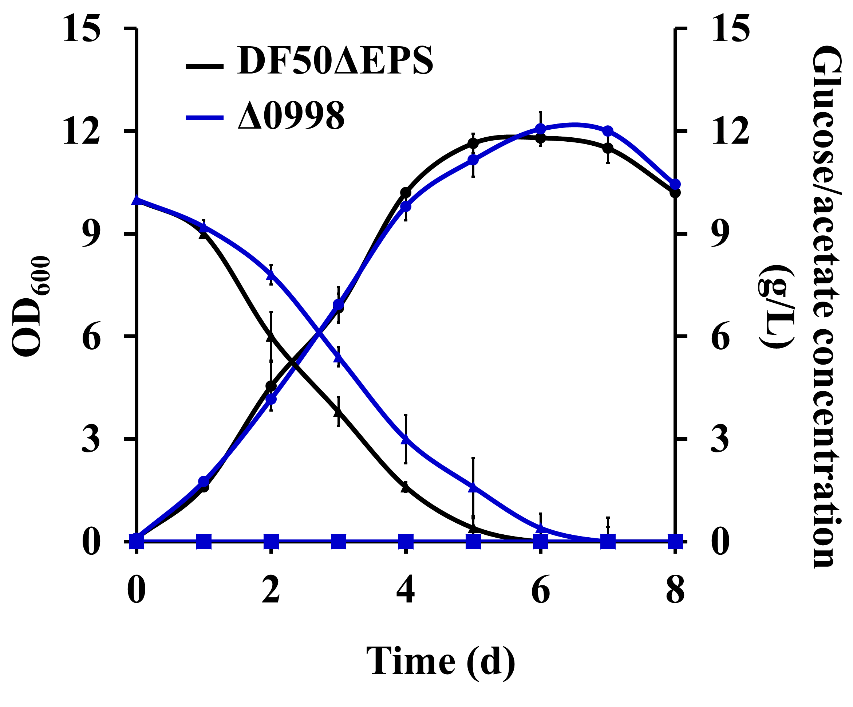


**Fig. S3 No detectable acetate overflow observed for *H. mediterranei* when using glucose as carbon source*.*** Growth (circles), glucose utilization (triangles), and acetate formation (squares) of DF50ΔEPS (black lines) and Δ0998 (blue lines) in 55 mM glucose containing medium. DF50ΔEPS represents the parent strain (positive control). Δ0998 represents HFX_0998 mutant of DF50ΔEPS. Data are expressed as mean ± SD, n = 3.


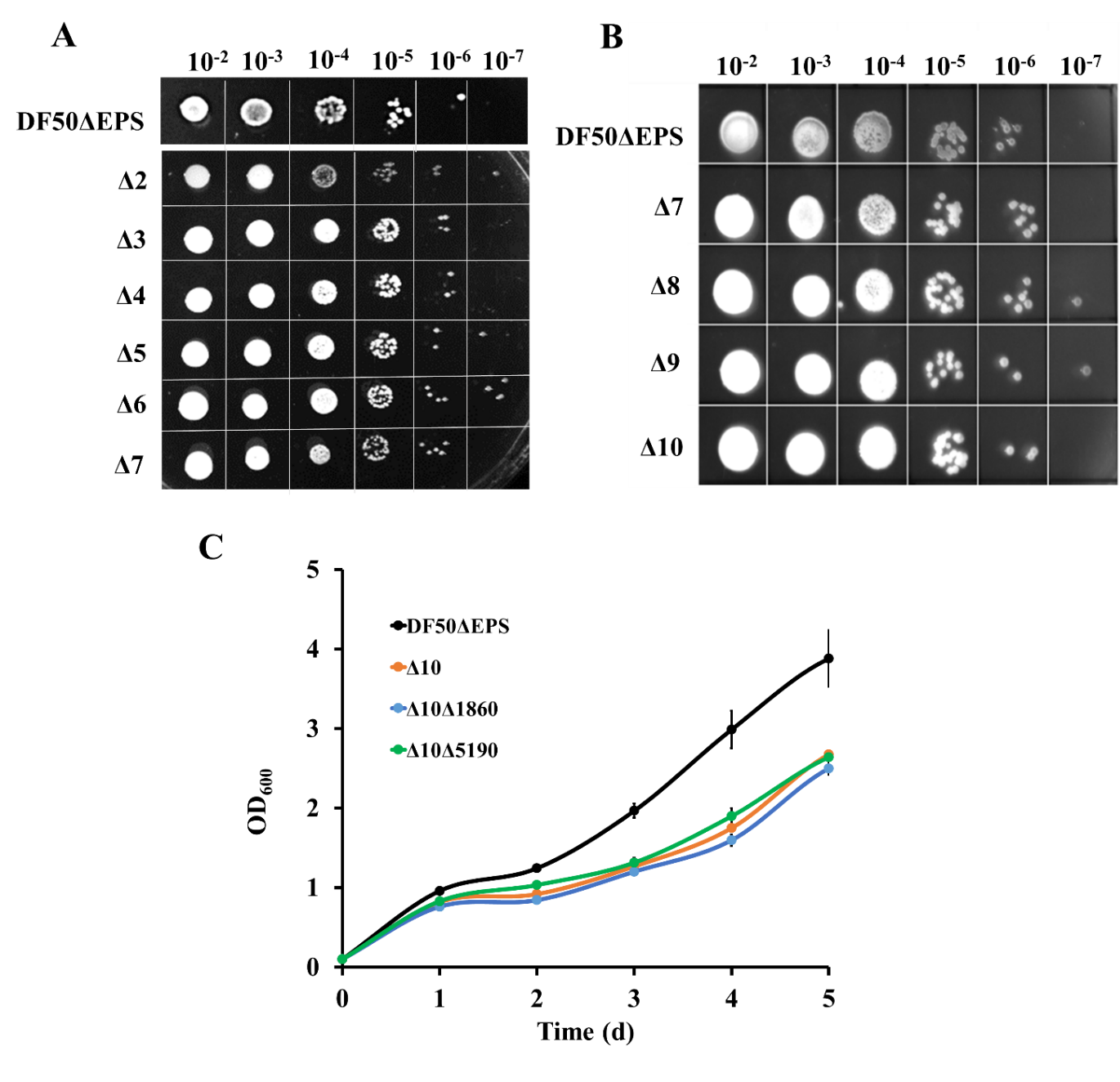


**Fig. S4 Growth defects in *acs* mutants of *H. mediterranei* on 0.12 M acetate*.*** (A) Spot assay of Δ2, Δ3, Δ4, Δ5, Δ6, and Δ7. Δ2, HFX_1643 mutant of Δ5131; Δ3, HFX_2150 mutant of Δ2; Δ4, HFX_1242 mutant of Δ3; Δ5, HFX_0870 mutant of Δ4; Δ6, HFX_5129 mutant of Δ5; and Δ7, HFX_0998 mutant of Δ6. (B) Spot assay of Δ7, Δ8, Δ9, and Δ10. Δ8, HFX_1451 mutant of Δ7; Δ9, HFX_4020 mutant of Δ8; Δ10, HFX_1837 mutant of Δ9. (C) Growth analysis of Δ10 (Orange line), Δ10Δ1860 (blue line), and Δ10Δ5190 (green line) in liquid medium. Δ10Δ1860, HFX_1860 mutant of Δ10; Δ10Δ5190, HFX_5190 mutant of Δ10. DF50ΔEPS (black line) represents positive control. Data are expressed as mean ± SD, n = 3 (C).


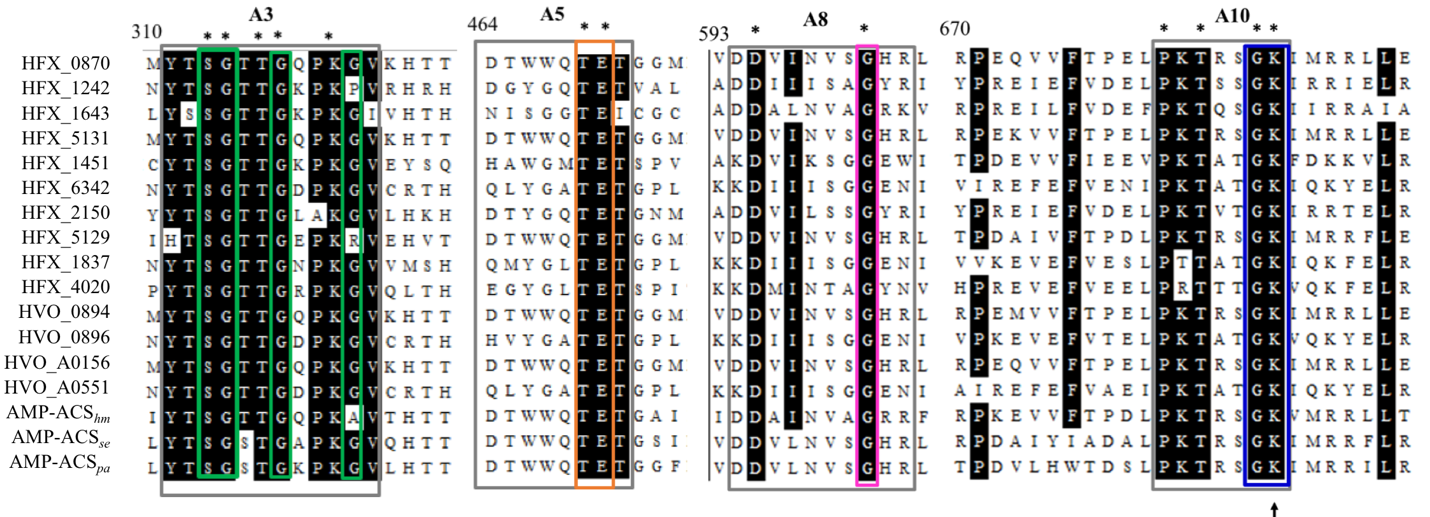


**Fig. S5 Amino acid sequence alignment of the ten candidate AMP-ACS of *H. mediterranei* with AMP-ACS from other archaea and bacteria*.*** Comparison of amino acid sequences of HFX_0870, HFX_1242, HFX_1643, HFX_5131, HFX_1451, HFX_6342, HFX_2150, HFX_5129, HFX_1837, and HFX_4020 with the AMP-ACS from *H. volcanii* (HVO_0894, HVO_0896, HVO_A0156, and HVO_A0551), *H. marismortui* (AMP-ACS*_hm_*), *Salmonella enterica* LT2 (AMP-ACS*_se_*), and *Pyrobaculum aerophilum* IM2 (AMP-ACS*_pa_*). Conserved SG rich loop (A3 region), TE dipeptide (A5 region), G residue (A8 region), GK dipeptide (A10 region) are indicated by green, orange, pink, and blue box, respectively. The highly conserved K residue is indicated by the black arrow.

**Reference**

1. Chen J, Mitra R, Zhang S, Zuo Z, Lin L, Zhao D, Xiang H, Han J. 2019. Unusual phosphoenolpyruvate (PEP) synthetase-like protein crucial to enhancement of polyhydroxyalkanoate accumulation in *Haloferax mediterranei* revealed by dissection of PEP-pyruvate interconversion mechanism. Appl Environ Microbiol 85:e00984-19.
2. Srere PA, Brazil H, Gonen L. 1963. The citrate condensing enzyme of pigeon breast muscle and moth flight muscle. Acta Chem Scand 17:129-134.
3. Schäfer T, Schönheit P. 1991. Pyruvate metabolism of the hyperthermophilic archaebacterium *Pyrococcus furiosus*: Acetate formation from acetyl-CoA and ATP synthesis are catalyzed by an acetyl-CoA synthetase (ADP forming). Arch Microbiol 155:366-377.
4. Zuo, ZQ, Xue Q, Zhou J, Zhao DH, Han J, Xiang H. 2018. Engineering *Haloferax mediterranei* as an efficient platform for high level production of lycopene. Front Microbiol 9:2893.
5. Zhao D, Cai L, Wu J, Li M, Liu H, Han J, Zhou J, Xiang H. 2013. Improving polyhydroxyalkanoate production by knocking out the genes involved in exopolysaccharide biosynthesis in *Haloferax mediterranei*. Appl Microbiol Biotechnol 97:3027-36.
6. Liu G, Hou J, Cai S, Zhao D, Cai L, Han J, Zhou J, Xiang H. 2015. A patatin-like protein associated with the polyhydroxyalkanoate (PHA) granules of *Haloferax mediterranei* acts as an efficient depolymerase in the degradation of native PHA. Appl Environ Microbiol 81:3029 –3038.
7. Liu H, Han J, Liu X, Zhou J, Xiang H. 2011. Development of *pyrF*-based gene knockout systems for genome-wide manipulation of the archaea *Haloferax mediterranei* and *Haloarcula hispanica*. J Genet Genomics 38: 261-269.
8. Cai S, Cai L, Liu H, Liu X, Han J, Zhou J, Xiang, H. 2012. Identification of the haloarchaeal phasin (PhaP) that functions in polyhydroxyalkanoate accumulation and granule formation in *Haloferax mediterranei*. Appl Environ Microbiol 78: 1946–1952.
9. Pinhal S, Ropers D, Geiselmann J, De Jong H. 2019. Acetate metabolism and the inhibition of bacterial growth by acetate. J Bacteriol 201:10-1128.
10. Dittrich CR, Bennett GN, San KY. 2005. Characterization of the acetate‐producing pathways in *Escherichia coli*. Biotechnol Prog 21:1062-1067.
11. Kumari S, Tishel R, Eisenbach M, Wolfe AJ. 1995. Cloning, characterization, and functional expression of *acs*, the gene which encodes acetyl coenzyme A synthetase in *Escherichia coli*. J Bacteriol 177:2878-2886.
12. Veit A, Rittmann D, Georgi T, Youn JW, Eikmanns BJ, Wendisch VF. 2009. Pathway identification combining metabolic flux and functional genomics analyses: acetate and propionate activation by *Corynebacterium glutamicum*. J Biotechnol 140:75-83.
13. Yasuda K, Jojima T, Suda M, Okino S, Inui M, Yukawa H. 2007. Analyses of the acetate-producing pathways in *Corynebacterium glutamicum* under oxygen-deprived conditions. Appl Microbiol Biotechnol 77:853-860.
14. Grundy FJ, Turinsky AJ, Henkin TM. 1994. Catabolite regulation of *Bacillus subtilis* acetate and acetoin utilization genes by CcpA. J Bacteriol 176:4527-4533.
15. Presecan-Siedel E, Galinier A, Longin R, Deutscher J, Danchin A, Glaser P, Martin-Verstraete I. 1999. Catabolite regulation of the *pta* gene as part of carbon flow pathways in *Bacillus subtilis*. J Bacteriol 181:6889-6897.
16. James KL, Ríos-Hernández LA, Wofford NQ, Mouttaki H, Sieber JR, Sheik CS, Nguyen HH, Yang Y, Xie Y, Erde J, Rohlin L. 2016. Pyrophosphate-dependent ATP formation from acetyl coenzyme A in *Syntrophus aciditrophicus*, a new twist on ATP formation. mBio 7:10-1128.
17. Zhang B, Lingga C, Bowman C, Hackmann TJ. 2021. A new pathway for forming acetate and synthesizing ATP during fermentation in bacteria. Appl Environ Microbiol 87:e02959-20.
18. Kwong WK, Zheng H, Moran NA. 2017. Convergent evolution of a modified, acetate-driven TCA cycle in bacteria. Nat Microbiol 2:1-3.
19. Mullins EA, Francois JA, Kappock TJ. 2008. A specialized citric acid cycle requiring succinyl-coenzyme A (CoA): acetate CoA-transferase (AarC) confers acetic acid resistance on the acidophile *Acetobacter aceti*. J Bacteriol 190:4933-4940.
20. Pettinato E, Böhnert P, Berg IA. 2022. Succinyl-CoA: acetate CoA-transferase functioning in the oxidative tricarboxylic acid cycle in *Desulfurella acetivorans*. Front Microbiol 13:1080142.
21. Schäfer T, Selig M, Schönheit P. 1993. Acetyl-CoA synthetase (ADP forming) in archaea, a novel enzyme involved in acetate formation and ATP synthesis. Arch Microbiol 159:72-83.
22. Rother M, Metcalf WW. 2004. Anaerobic growth of *Methanosarcina acetivorans* C2A on carbon monoxide: an unusual way of life for a methanogenic archaeon. Proc Natl Acad Sci 101: 16929-16934.
23. Welte C, Kröninger L, Deppenmeier U. 2014. Experimental evidence of an acetate transporter protein and characterization of acetate activation in aceticlastic methanogenesis of *Methanosarcina mazei*. FEMS Microbiol Lett 359:147-153.
24. Kuprat T, Johnsen U, Ortjohann, M, Schönheit, P. 2020. Acetate metabolism in archaea: characterization of an acetate transporter and of enzymes involved in acetate activation and gluconeogenesis in *Haloferax volcanii*. Front Microbiol 11:604926.
25. Kuprat T, Ortjohann M, Johnsen U, Schönheit P. 2021. Glucose metabolism and acetate switch in Archaea: The enzymes in *Haloferax volcanii*. J Bacteriol 203:10-1128.
26. Bräsen C, Schönheit P. 2005. AMP-forming acetyl-CoA synthetase from the extremely halophilic archaeon *Haloarcula marismortui*: purification, identification and expression of the encoding gene, and phylogenetic affiliation. Extremophiles 9:355-365.
27. Bräsen C, Schönheit P. 2004. Unusual ADP-forming acetyl-coenzyme A synthetases from the mesophilic halophilic euryarchaeon *Haloarcula marismortui* and from the hyperthermophilic crenarchaeon *Pyrobaculum aerophilum*. Arch Microbiol 182:277-287.
